# Optimal Dietary Vitamin A Fortification Improves Growth Performance, Nutrient Metabolism, Antioxidant Capacity, Immune Response, and Histomorphology in Intensively Cultured *Litopenaeus vannamei*

**DOI:** 10.3390/ani16192978

**Published:** 2026-09-22

**Authors:** Di Liu, Lu Zhang, Xiaoru Chen, Mingjie Chen, Yuelan Wen, Lixian Li, Wencen Peng, Hao Chen, Beiping Tan, Shuyan Chi

**Affiliations:** 1 Laboratory of Aquatic Animal Nutrition and Feed, College of Fisheries, Guangdong Ocean University, Zhanjiang 524088, China; liudi_gdou@163.com (D.L.);; 2 Key Laboratory of Aquatic Nutrition and Smart Farming, Ministry of Agriculture and Rural Affairs, Chengdu 610041, China; zhangl21@tongwei.com (L.Z.);; 3 Aquatic Health and Intelligent Aquaculture Key Laboratory of Sichuan Province, Chengdu 610041, China; 4 Tongwei Agricultural Development Co., Ltd., Chengdu 610041, China

**Keywords:** vitamin A, *Litopenaeus vannamei*, growth performance, antioxidant, nutrient metabolism, immunity

## Abstract

Vitamin A (VA) functions as a vital micronutrient supporting growth, immune competence, and stress tolerance in shrimp, though its requirement under industrial high-density rearing conditions has not been established. This study assessed how graded dietary VA levels affect growth, nutrient metabolism, antioxidant capacity, immune function, and hepatopancreas structure in *Litopenaeus vannamei* reared at 720 shrimp/m^3^. Five experimental diets were formulated with graded VA supplementation at 0 (A0, control), 3000 (A1), 6000 (A2), 12,000 (A3), and 24,000 IU/kg (A4). During an 8-week feeding trial, the weight gain rate (WGR) and specific growth rate (SGR) increased and subsequently decreased with rising dietary VA, while the feed conversion ratio (FCR) showed the opposite trend; the A3 group exhibited optimal performance. In the A2 and A3 groups, trypsin, lipase, and amylase activities were enhanced, accompanied by the upregulation of protein synthesis-related genes (*tor*, *s6k*) and fatty acid oxidation-related genes (*cpt-1*, *aco*), as well as the downregulation of lipogenic genes (*acc*, *fas*) (*p* < 0.05), which resulted in an elevated whole-body crude protein content (*p* < 0.05). In the hepatopancreas, activities and transcript levels of SOD, GSH-Px, and PO increased, the MDA content decreased, and histological status improved. Immune-related genes (*tlr4*, *myd88*, *nf-κB*, *imd*, *relish*, *lzm*, *crustins*) were upregulated, whereas *hsp70* and *aif* were downregulated (*p* < 0.05), suggesting enhanced innate immune potential and reduced stress-associated responses. Under the high-density culture system in this study, *L. vannamei* exhibited improved growth performance when the dietary VA level was 12,647 IU/kg. Based on weight gain results, the dietary VA concentration required for maximum shrimp growth under this stocking density was estimated to be 22,800 IU/kg. This value is model-derived rather than an established dietary requirement and is subject to uncertainty, requiring further experimental validation.

## Simple Summary

*Litopenaeus vannamei* is an economically important farmed crustacean with rapid growth and strong adaptability. Intensive high-density industraial aquaculture has gradually replaced traditional pond culture to meet industrial upgrading demands, yet whether high-density stress alters shrimp nutritional requirements remains poorly understood. In this 8-week indoor industrial rearing trial at a stocking density of 720 individuals/m^3^, we comprehensively evaluated shrimp growth performance, serum biochemistry, digestive and antioxidant enzyme activities, hepatopancreatic histomorphology, and immune gene expression under graded dietary VA supplementation. Moderate VA improved digestive capacity, facilitated protein deposition and lipid catabolism, enhanced antioxidant defense and innate immunity, and relieved stress-induced hepatopancreatic damage. Excessive VA exerted adverse effects on metabolism and tissue structure. The optimal vitamin A level determined under the present high-density condition is higher than that reported under conventional low-density conditions, suggesting that high-density culture may elevate the vitamin A requirement.

## 1. Introduction

*Litopenaeus vannamei* ranks as the top farmed crustacean in China, with its production contributing more than 80% of the national total farmed shrimp yield by 2024 [1]. As the industry continues to expand, the traditional pond culture system, owing to its high susceptibility to environmental fluctuations, low yield per unit area, and limited resource-use efficiency, can no longer meet the requirements of high-quality development in modern shrimp aquaculture. The industrialized aquaculture model, characterized by a high stocking density, intensification, and controllability, such as temperature control, aeration, water purification, and smart management, has expanded rapidly [2,3,4]. Relative to conventional ponds and elevated ponds, the industrial high-density model has gained wide adoption on the strength of its high density, intensification, and intelligent controllability; stocking densities can reach 500–1000 individuals/m^3^ or higher [3,5,6]. However, in the late culture period, such high-density conditions readily generate multiple environmental stressors, such as restricted living space, ammonia-nitrogen and nitrite accumulation from uneaten feed and metabolic waste, and dissolved oxygen depletion, which disturb shrimp metabolic homeostasis and accelerate the depletion of fat-soluble antioxidant vitamins such as VA [7,8,9,10]. Existing research shows that VA engages in a bidirectional interaction with the principal environmental stresses in shrimp culture [11]. Combined hypoxic–ammonia stress can directly perturb the retinol metabolic pathway in shrimp and upset VA homeostasis [12]. Adequate dietary VA markedly alleviated the tissue injury, oxidative stress, and inflammation triggered by acute hypoxia in grass carp (*Ctenopharyngodon idella*) [13,14] and raised survival and stress tolerance to ammonia-nitrogen stress in Pacific white shrimp and Chinese mitten crab (*Eriocheir sinensis*) [15,16]. With respect to high-density crowding, VA enhanced the stress resistance of organisms by upregulating the activities of antioxidant enzymes and maintaining the integrity of mucosal barriers [11,17].

VA serves critical functions including vision formation, embryonic development, cell differentiation, and tissue repair [18,19]. Optimal VA levels can enhance growth, improve feed utilization efficiency and specific growth rate, and are involved in immune regulation and antioxidant defense in aquatic animals [20,21,22]. When the dietary VA supply is insufficient, aquatic animals frequently exhibit growth retardation, increased mortality, tissue hemorrhage, skeletal and appendage developmental abnormalities, and reduced vitality, severely compromising aquaculture profitability [23,24].

VA is emulsified into bile-salt micelles, packaged into chylomicron-like/caroteno lipoprotein particles, and deposited in the hepatopancreas [25]. Mechanistically, how crustaceans handle dietary VA remains far less defined than in the well-characterized vertebrate system. Instead, *L. vannamei* converts dietary β-carotene and astaxanthin into all-trans-retinol, all-trans-retinal, and 13-cis isomers that accumulate in the hepatopancreas, ovary, and eyestalk and are carried through the hemolymph by carotenolipoproteins [26]. These retinoids are then plausibly taken up via lipoprotein receptor-mediated endocytosis and passive partitioning into hepatopancreatic cells [25], and a cellular retinoic acid/retinol binding protein has been characterized in the shrimp hepatopancreas [27]. This crustacean-specific carotenoid–retinoid axis sustains innate immunity, antioxidant defense, and gonadal maturation–precisely the endpoints compromised by ammonia, nitrite, and hypoxia [11,28].

VA retention levels in the body would affect the lipid metabolic status and regulate lipogenesis through fatty acid synthase (FAS), acetyl-CoA carboxylase (ACC), glycerol-3-phosphate dehydrogenase (GPDH), and peroxisome proliferator-activated receptor α (PPARα) [29,30]. Studies have shown that *L. vannamei* remodeled hepatopancreatic metabolism through the AMPK/PI3K–Akt pathway under prolonged high-density stress [31]; ammonia stress (6–64 mg/L) upregulated gene expression levels of FAS, ACC, and carnitine palmitoyltransferase-1 (CPT-1) and caused excessive deposition of triglycerides [32], directly disrupting the lipogenic machinery that sustains VA transport. Consequently, the chronic stress imposed by high-density culture disrupts the VA–lipid interplay at multiple nodes.

At present, most existing data on the VA requirement of *L. vannamei* have been derived from traditional earthen ponds or low-density culture conditions. Accordingly, does such a dosage also meet the growth requirements of shrimp under high-density farming conditions? The present study used an industrial high-density culture model for *L. vannamei* to investigate the VA regulated growth performance and health status. The specific objectives were to: (1) determine the optimal dietary VA supplementation level for *L. vannamei* under industrial high-density culture based on growth performance, feed utilization, and survival rate; (2) elucidate the regulatory mechanisms of graded VA levels on hepatopancreatic lipolysis and lipogenesis, as well as antioxidant and immune capacities. The results of this study are expected to provide a theoretical basis for the formulation of VA-specific compound feeds for industrial aquaculture.

## 2. Materials and Methods

### 2.1. Preparation of Experimental Feed

VA acetate (active content: 500,000 IU/g, Zhejiang Medicine Co., Ltd., Shaoxing, China) served as the VA source in the present study. The vitamin premixes containing the different levels of VA required for the experiment were manufactured by Qingdao Master Biotech Co., Ltd. (Qingdao, China) to ensure an accurate dosage and uniform distribution. These tailor-made premixes were incorporated into the basal diet to formulate experimental diets with gradient VA levels. Five experimental diets with equal nitrogen and lipid contents were prepared, with A0 as the control and A1–A4 as the four VA gradient groups, each with four replicates. The VA supplementation levels were 0 (A0, control), 3000 (A1), 6000 (A2), 12,000 (A3), and 24,000 IU/kg (A4). All diets were processed by the shrimp feed production line of Qingdao Qihao Bio-tech Co., Ltd. (Qingdao, China) and pelleted into 1.0-mm and 1.5-mm pellets. The detailed formulation is shown in Table 1. After processing, the diets were stored in a cool, light-protected warehouse at the industrialized aquaculture facility until use. The actual measured VA contents of the five experimental diets were 868, 3750, 6870, 12,647, and 24,758 IU/kg. Each experimental diet was analyzed in triplicate for nutritional composition determination. All dose–response analyses in the present study were performed based on the determined actual dietary VA concentrations, rather than the designed supplemental levels, to ensure accurate and reliable estimation of vitamin A requirement.

### 2.2. Experimental Animals and Rearing Conditions

This feeding trial was conducted at the industrialized aquaculture workshop of Bohai Fishery (Binzhou) Co., Ltd. (Binzhou, China) (Figure 1). Juvenile *L. vannamei* were obtained from Qingdao Hairen Aquatic Breeding Technology Co., Ltd (Qingdao, China). and were uniform in size and healthy. Prior to the trial, shrimp were communally reared to the target size and then randomly assigned to 20 experimental tanks, with an initial mean body weight (IBW) of 0.26 ± 0.01 g. The experimental tanks were circular, measuring 6 m in diameter and 1 m in water depth, holding 28 m^3^ of the effective water volume, with shrimp stocked at a density of 720 individuals/m^3^. The trial comprised five treatment groups, each with four replicates. The culture period lasted 8 weeks. Shrimp were fed six times daily at 4-h intervals (01:00, 05:00, 09:00, 13:00, 17:00, and 21:00). The initial daily feeding level was 6% of shrimp biomass. The daily ration was adjusted: the feed amount increased by 3% if feed was consumed within 2 h; otherwise, the feeding rate remained unchanged. Uneaten feed was not collected or quantified. Tank bottoms were cleaned at 10:00 each morning, and the water exchange rate was 40–70% of the total tank volume per day, adjusted based on the shrimp biomass and feeding rate. Water quality parameters were monitored daily: temperature 28.5 ± 1.2 °C, dissolved oxygen ≥ 5.0 mg/L, pH 7.9 ± 0.3, total ammonia nitrogen ≤ 0.10 mg/L, and nitrite-N ≤ 0.01 mg/L. Water-quality parameters did not differ significantly among treatment tanks.

### 2.3. Sample Collection

At the end of the feeding trial, all *L. vannamei* were fasted for 24 h. Approximately 1.0 kg of shrimp were randomly collected from each tank, counted, and returned to the tank; mean individual body weight and total weight were recorded for the calculation of final body weight (FBW), weight gain rate (WGR), specific growth rate (SGR), and feed conversion ratio (FCR). All subsequent samples were taken from this source. Four shrimp were randomly selected from each tank, dried, and pooled for body composition determination (one value was obtained per tank; *n* = 4 per treatment). Six shrimp were randomly selected from each tank, and hemolymph was collected in 1.5 mL centrifuge tubes and stored overnight at 4 °C. The samples were then centrifuged at 3500 r/min for 10 min at 4 °C, and the supernatant (serum) was collected. Serum from the six shrimps was pooled into one composite sample per tank for subsequent biochemical analysis. Another six shrimps were randomly selected from each tank and rapidly dissected to collect the hepatopancreas, which was immediately snap-frozen in liquid nitrogen and stored at −80 °C. The hepatopancreas from every two shrimps were pooled to prepare one composite sample, yielding three composite samples per tank for enzyme activity analysis. Three shrimps were randomly selected from each tank, and a small piece of hepatopancreatic tissue (approximately soybean-sized) was excised and immediately placed in a 2 mL RNase-free centrifuge tube containing RNA preservation solution (−80 °C for storage). The three shrimp from each tank were treated as separate samples; total RNA was individually extracted, reverse-transcribed, and subjected to qPCR. Two shrimp per tank were randomly sampled; hepatopancreases were dissected and immediately fixed in 4% paraformaldehyde solution (4% paraformaldehyde in PBS, pH 7.2–7.4) for 24 h for paraffin sectioning and hematoxylin and eosin (H&E) staining.

### 2.4. Proximate Composition Analysis

Proximate composition (moisture, crude protein, crude lipid, and ash) of diets and whole-shrimp samples was determined following the standard methods of the Association of Official Analytical Chemists (AOAC) [33]. The whole-body composition was determined as follows: moisture by oven-drying at 105 °C to constant weight (wet-weight basis); crude protein by Kjeldahl method (N × 6.25, dry-matter basis); crude lipid by ether extraction (dry-matter basis); and ash by combustion at 550 °C for 6 h (dry-matter basis).

Diet samples were ground through a 1-mm sieve, and the determination of VA in the experimental diets was performed under dim light to prevent photo-oxidative degradation of VA. VA was determined by saponification-HPLC (Agilent 1200-HPLC, Santa Clara, CA, USA) following GB/T 17817-2024 [34]. Briefly, ~10 g of feed sample (0.001 g precision) was saponified in a boiling water bath for 30 min with ascorbic acid and BHT as antioxidants. After saponification, the analytes were extracted with light petroleum ether. The organic phase was washed to neutral, dehydrated with anhydrous sodium sulfate, concentrated under reduced pressure at low temperature, re-dissolved in methanol, and filtered through a 0.22 μm organic membrane. Mobile phase: methanol-water; flow rate: 1.0 mL/min; column (C18 column, 4.6 mm × 250 mm, 5 μm) temperature: 35 °C; detection wavelength: 325 nm; injection volume: 10 μL. External standard calibration was used for quantification. Each sample was pretreated independently and analyzed in triplicate, and results were expressed as mean values (IU/kg).

### 2.5. Serum Biochemical Indices

Serum total cholesterol (TCHO), triglycerides (TG), high-density lipoprotein cholesterol (HDL-C), and low-density lipoprotein cholesterol (LDL-C) were measured using commercial kits (Nanjing Jiancheng Bioengineering Institute, Nanjing, China) following the manufacturer’s protocols, with concentrations calculated from standard curves or provided formulas.

### 2.6. Hepatopancreas Enzyme Activity Assays

Hepatopancreas samples were homogenized in pre-chilled physiological saline (0.86% NaCl) at a mass-to-volume ratio of 1:9 (w:v) in an ice bath to prepare 10% tissue homogenates. The homogenate was centrifuged at 4 °C and 3500 rpm for 10 min, and the supernatant was collected for analysis. Phenoloxidase (PO), superoxide dismutase (SOD), and glutathione peroxidase (GSH-Px) activities and the malondialdehyde (MDA) content were measured using commercial kits (Nanjing Jiancheng Bioengineering Institute) according to the manufacturer’s instructions. Hepatopancreas lipase, trypsin, and amylase (AMY) activities were measured using ELISA kits (Shanghai Enzyme-linked Biotechnology Co., Ltd., Shanghai, China), with absorbance read at 450 nm. The protein concentration of the samples was determined by the Coomassie brilliant blue method.

### 2.7. RT-qPCR Analysis

Three hepatopancreas samples from individual shrimp per tank were analyzed with three technical replicates each. Total RNA of the hepatopancreas was extracted using TRIzol reagent (TransGen Biotech Co., Ltd.; Cat. No. ET101-01; Beijing, China). RNA integrity was verified by 1.5% agarose gel electrophoresis (28S:18S rRNA ≥ 1.5) and spectrophotometry (A260/A280 = 1.8–2.0; NanoDrop 2000, Thermo Fisher; Waltham, MA, USA). First-strand cDNA was synthesized using the HiScript III RT SuperMix for qPCR kit (Vazyme; Cat. No. R223-01; Nanjing, China). qPCR was performed on a QuantStudio 5 system (Applied Biosystems) using ChamQ Universal SYBR qPCR Master Mix (Vazyme; Cat. No. Q711-02). The total reaction volume was 10 μL, comprising 5.0 μL of 2× Master Mix, 0.4 μL each of forward and reverse primers (10 μmol/L), 1.0 μL of cDNA template, and 3.2 μL of RNase-free water. Thermal profile: 95 °C/30 s; 40 cycles of 95 °C/5 s, 60 °C/30 s. Melt curve analysis (60–95 °C) confirmed primer specificity (single peak). Amplification efficiencies (92.3–108.5%; R^2^ ≥ 0.992) were reported in Table 2. Gene annotations were verified against NCBI and UniProt databases; gene symbols follow shrimp nomenclature conventions. LvEF1α was used as the reference gene, and relative gene expression was calculated using the 2^−ΔΔCt^ method. Gene accession numbers and primer sequences are listed in Table 2.

### 2.8. Hepatopancreas Histomorphology

Hepatopancreas tissues of *L. vannamei* were carefully dissected and fixed in 4% paraformaldehyde in PBS at room temperature for 24 h. The tissues were then processed through graded ethanol dehydration, xylene clearing, and paraffin embedding, sectioned at 5 μm, deparaffinized, and stained with hematoxylin and eosin (H&E). Tissue section preparation was performed by Servicebio (Wuhan, China). Sections were examined under a light microscope (Eclipse 80i, Nikon, Tokyo, Japan). For hepatopancreatic histological observation, 2 shrimp were randomly sampled from each tank, with a total of 8 shrimp per treatment, and 3 non-overlapping sections were prepared per individual shrimp. All histological sections were examined blindly to ensure objective and reliable results.

### 2.9. Calculation Formulas

Weight gain rate (WGR, %) = 100 × (final mean body weight − initial mean body weight)/initial mean body weight.

Specific growth rate (SGR, %/d) = 100 × (ln final mean body weight − ln initial mean body weight)/feeding days.

Feed conversion ratio (FCR) = total feed intake/(final total biomass − initial total biomass).

### 2.10. Statistical Analysis

All analyses of group differences and Pearson correlation were performed using SPSS 20.0 (SPSS Inc., Chicago, IL, USA). Levene’s test was used to verify the homogeneity of variances, followed by one-way ANOVA. When significant differences were detected (*p* < 0.05), Tukey’s multiple range test was applied for post hoc pairwise comparisons. The significance level was set at *p* < 0.05. Regression equation fitting was conducted with Origin 2026 (OriginLab Corporation, Northampton, MA, USA), and bar charts were generated using GraphPad Prism 10.6.0 (GraphPad Software, LLC, Boston, MA, USA).

## 3. Results

### 3.1. Growth Performance

#### 3.1.1. Growth Performance of Shrimp

Table 3 shows that after the 8-week trial, the final body weight (FBW) was significantly affected by dietary VA levels (*p* < 0.05). The A3 group recorded the highest FBW, WGR, and SGR, exceeding all other treatments (*p* < 0.05). A4 outperformed A0, A1, and A2 in FBW, WGR, and SGR (*p* < 0.05) but remained inferior to A3 (*p* < 0.05). FCR declined initially and rebounded modestly with rising VA levels. A3 produced the lowest FCR, which was significantly below all other groups (*p* < 0.05); A4 yielded a lower FCR than A0, A1, and A2 (*p* < 0.05) but exceeded A3 (*p* < 0.05).

As shown in Figure 2, quadratic regression analysis was performed based on weight gain rate. The regression equation was $y=-1.0\times 10^{-6}x^{2}+0.0456x+1870.8$, with R^2^ = 0.8463. According to the calculation, the dietary vitamin A level for maximum weight gain was 22,800 IU/kg diet. Notably, the group supplemented with 12,647 IU/kg VA achieved optimal growth performance in this trial. The 22,800 IU/kg value, estimated by second-order regression analysis based on weight gain, represents a model-predicted result instead of a definitive dietary nutrient requirement and carries uncertainty, awaiting further experimental validation.

#### 3.1.2. Whole-Body Composition of Shrimp

As displayed in Table 4, the whole-body moisture content in A1 and A3 surpassed that of the other groups (*p* < 0.05). A3 had a higher whole-body crude protein than A1 (*p* < 0.05), while the remaining groups did not differ significantly (*p* > 0.05). A4 had a higher whole-body crude lipid content than all other groups (*p* < 0.05); A0, A2, and A3 were not significantly different (*p* > 0.05). A2 and A3 had a higher whole-body crude ash than the other groups (*p* < 0.05), whereas A1 was lower than all other groups (*p* < 0.05).

### 3.2. Hepatopancreas Histomorphology of Shrimp

As shown in Figure 3, in A0, hepatopancreatic tubules were loosely arranged with irregular lumen morphology; glandular epithelial cells (B cells and R cells) were sparsely distributed with indistinct morphology, and extensive vacuolization was observed within the tubular lumens, indicating poor tissue integrity. Compared with group A0, the hepatopancreas structure in group A1 showed improvement, with more regular tubule morphology and clearer B and R cell differentiation, although slight vacuolization was still present. Group A2 showed further improvement, with orderly arranged tubules, distinct cell boundaries, uniform distribution of digestive and absorptive cells, intact lumens, and tissue integrity significantly superior to that of groups A0 and A1. The A3 group exhibited the best physiological state and functional integrity of the hepatopancreas, with densely packed tubules, well-defined lumen contours, plump cells with uniform nuclear staining, and virtually no vacuolization. In group A4, structural degeneration of the hepatopancreas was observed, including loose glandular arrangement, localized vacuolization, and mildly disordered tissue structure.

### 3.3. Digestive Enzyme Activities in the Hepatopancreas of Shrimp

Figure 4 illustrates that the trypsin activity rose initially and then fell with rising dietary VA. Trypsin peaked in A2, exceeding A0 and A1 (*p* < 0.05), but was not significantly different from A3 or A4 (*p* > 0.05). Lipase peaked in A2 and A3, which did not differ from each other (*p* > 0.05) but exceeded that in all other treatments (*p* < 0.05); A4 came next, surpassing A0 and A1 *(p* < 0.05). AMY activity was also significantly affected by VA levels (*p* < 0.05), showing an overall pattern of initial increase, subsequent decrease, and then increase again. AMY peaked in A3, exceeding A0, A2, and A4 (*p* < 0.05) but not A1 (*p* > 0.05); A0, A2, and A4 did not differ (*p* > 0.05).

### 3.4. Lipid Metabolism Capacity of Shrimp

#### 3.4.1. Serum Biochemical Indices of Shrimp

Serum TG and TCHO levels declined progressively with rising VA (Figure 5). TG levels in A0, A1, and A2 were comparably elevated, without significant differences (*p* > 0.05), but all exceeded A3 and A4 (*p* < 0.05). TCHO was highest in A0 and lowest in A3 and A4, with A3 and A4 not differing significantly (*p* > 0.05). HDL-C rose then declined, with A2 and A3 surpassing the other groups (*p* < 0.05). LDL-C peaked in A1 and bottomed in A3, with A2 and A3 below A0 and A1 (*p* < 0.05).

#### 3.4.2. Effects on Key Gene Expression Levels in Lipid Metabolism

Figure 6 shows that *cpt-1* expression rose and then fell with rising VA. *cpt-1* expression in A1–A4 all exceeded A0 (*p* < 0.05); A3 was the highest, and A4 was significantly below A3 (*p* < 0.05). *aco* expression trended upward with rising VA; A2, A3, and A4 did not differ (*p* > 0.05) but all exceeded A0 and A1 (*p* < 0.05), with A3 the highest. acc expression in all treatment groups fell below A0 (*p* < 0.05), with A3 the lowest, below all other groups (*p* < 0.05). *fas* expression paralleled that of acc, with all treatments below A0 and A3 the lowest (*p* < 0.05).

### 3.5. Effects on Key Gene Expression Levels in Protein Synthesis

Figure 7 reveals that *s6k* expression peaked in A3, exceeding all other treatments (*p* < 0.05). *s6k* in A4 dropped below A3 but stayed above A0, A1, and A2 (*p* < 0.05). *tor* expression trended upward initially before declining. *tor* peaked in A3, exceeding all other groups (*p* < 0.05); A2 and A4 followed, not differing from each other (*p* > 0.05), but both were above A1 and A0 (*p* < 0.05); *tor* was lowest in A0. *4e-bp* expression was highest in A2 and was not significantly different from A3 (*p* > 0.05) but was above A0, A1, and A4 (*p* < 0.05).

### 3.6. Antioxidant Capacity

#### 3.6.1. Antioxidant Enzyme Activities in the Hepatopancreas of Shrimp

As shown in Figure 8, PO activity rose, then slightly declined with rising VA. PO peaked in A3, surpassing all other groups (*p* < 0.05); A4 followed, with A0 the lowest. GSH-Px mirrored this pattern, peaking in A3, above A0, A1, and A4 (*p* < 0.05) but not significantly different from A2 (*p* > 0.05); A0, A1, and A4 did not differ (*p* > 0.05). SOD in A1–A4 was comparable (*p* > 0.05) but uniformly above A0 (*p* < 0.05). The MDA content trended downward with rising VA. MDA was lowest in A3, below all other groups (*p* < 0.05); A4 was lower than A0, A1, and A2 but exceeded A3 (*p* < 0.05). A0 had the highest MDA, with A1 and A2 intermediate and all three differing significantly (*p* < 0.05).

#### 3.6.2. Antioxidant Enzyme Gene Regulation in the Hepatopancreas

*sod* in all treatments exceeded A0 (*p* < 0.05); A3 and A4 were above A0, A1, and A2 (*p* < 0.05), with A3 and A4 not differing *(p* > 0.05). *po* expression rose then declined. *po* peaked in A3 and was above all other groups (*p* < 0.05); A1, A2, and A4 all exceeded A0 (*p* < 0.05), with the three not differing (*p* > 0.05). *gshpx* expression trended upward with rising VA. *gshpx* peaked in A4, above A0, A1, and A2 (*p* < 0.05), but was not significantly different from A3 (*p* > 0.05) (Figure 9).

### 3.7. Key Gene Expression Levels in Hepatopancreas Immune Regulation

Figure 10 shows that *rxr* expression in A2 and A3 exceeded all other groups (*p* < 0.05), with A2 and A3 not differing (*p* > 0.05). *tlr4* was elevated in A3 and A4, which did not differ (*p* > 0.05), but were above all other groups. *myd88* expression trended upward with rising VA; all treatments exceeded A0, with A3 the highest and above all others (*p* < 0.05). *nf-κB* in A2 and A3 exceeded A0 and A1 (*p* < 0.05). *hsp70* declined initially then rebounded with rising VA; A2 and A3 were below all other groups (*p* < 0.05). *aif* expression mirrored hsp70, with A3 the lowest and below all others (*p* < 0.05). *imd* in A2 and A3 exceeded all others (*p* < 0.05), with A2 and A3 not differing (*p* > 0.05). *relish* in A2 and A3 was elevated, above A0, A1, and A4 (*p* < 0.05), with A2 and A3 not differing (*p* > 0.05); *relish* was lowest in A4. *lzm* expression trended upward overall. A1 was comparable to A0 (*p* > 0.05); A2 was comparable to A3 and A4 (*p* > 0.05) but exceeded A0 (*p* < 0.05). *crustins* in all treatments exceeded A0 (*p* < 0.05) and peaked in A3; A1, A2, and A4 did not differ (*p* > 0.05).

### 3.8. Correlation Analysis of Growth Performance, Lipid Metabolism, Immune-Antioxidant Indices, and Their Related Gene Expression

Figure 11 shows the correlation heatmap between growth performance indices (WGR, FCR) and mTOR pathway genes (*s6k*, *tor*, *4e-bp*). WGR was extremely significantly negatively correlated with FCR (*p* < 0.01). WGR exhibited extremely significant positive correlations with *s6k* and *tor* (*p* < 0.01), whereas no significant correlation was observed between WGR and *4e-bp* (*p* > 0.05). FCR was extremely significantly negatively correlated with *s6k* and *tor* (*p* < 0.01), and showed a significant negative correlation with *4e-bp* (*p* < 0.05). Collectively, the high expression of key mTOR-pathway genes was closely associated with improved growth performance and a reduced feed coefficient in shrimp.

Figure 11B displays the Pearson correlations among serum lipid parameters (TG, TCHO, HDL-C, LDL-C) and lipid-metabolism-related genes (*cpt-1*, *aco*, *acc*, *fas*). Serum TG was extremely significantly positively correlated with TCHO and LDL-C (*p* < 0.01). TG had an extremely significant negative correlation with *aco* (*p* < 0.01) and an extremely significant positive correlation with *fas* (*p* < 0.01). TCHO was extremely significantly negatively correlated with *aco* (*p* < 0.01) and displayed a significant positive correlation with *acc* (*p* < 0.05) and an extremely significant positive correlation with *fas* (*p* < 0.01). HDL-C was extremely significantly positively correlated with *cpt-1* (*p* < 0.01), and significantly positively correlated with *aco* and *fas* (*p* < 0.05). LDL-C was significantly positively correlated with *fas* (*p* < 0.05). The upregulated expression of lipogenic genes (*acc*, *fas*) was accompanied by elevated serum TG and TCHO levels, while *aco* expression changed in the opposite direction to serum lipid levels, indicating that alterations in gene expression exert strong regulatory effects on host lipid metabolic status.

The figure illustrates the correlation heatmap of immune-antioxidant indices (PO, SOD, GSH-Px) and related genes (*po*, *sod*, *gshpx*, *rxr*, *myd88*, *imd*, *hsp70*, *aif*). PO activity was significantly positively correlated with SOD and GSH-Px activities (*p* < 0.05), extremely significantly positively correlated with *po*, *sod*, and *myd88* (*p* < 0.01), and significantly positively correlated with *imd* (*p* < 0.05). PO activity showed extremely significant negative correlations with *hsp70* and *aif* (*p* < 0.01). GSH-Px activity was significantly positively correlated with *po*, *rxr*, and *imd* (*p* < 0.05), extremely significantly negatively correlated with *hsp70* (*p* < 0.01), and significantly negatively correlated with *aif* (*p* < 0.05). SOD activity was significantly negatively correlated with *hsp70* and *aif* (*p* < 0.05). The immune gene *myd88* was extremely significantly positively correlated with *po*, *sod* and *gshpx* (*p* < 0.01). The stress- and apoptosis-related genes *hsp70* and *aif* were extremely significantly positively correlated with each other (*p* < 0.01). These results suggest that the expression of *hsp70* and *aif* is suppressed when immune-antioxidant capacity is enhanced.

## 4. Discussion

The industrialized high-density aquaculture model is characterized by a high stocking density, high feeding rates, and limited water exchange. Although this system can significantly increase the yield per unit area, it also leads to elevated ammonia nitrogen, nitrite, and organic load in the culture water, subjecting the animals to chronic stress and thereby impairing nutrient digestion and absorption as well as growth and development [35,36].

### 4.1. Growth Performance

Early studies found that the VA requirement of *L. vannamei* under laboratory conditions was approximately 4000–10,000 IU/kg [37,38], and the optimal VA requirement of juvenile *Penaeus monodon* was approximately 8400 IU/kg. Additional studies have indicated that dietary VA can enhance the physiological and biochemical status of *L. vannamei* but do not provide definitive requirement values [26,39]. A high stocking density (≥300 individuals/m^3^) activates the adenosine 5′-monophosphate-activated protein kinase (AMPK) and phosphatidylinositol 3-kinase-protein kinase B (PI3K-Akt) signaling pathways in the hepatopancreas of *L. vannamei*, reduces lysozyme and catalase activities [31], and imposes persistent metabolic and immune burdens. Furthermore, high stocking density also decreases feed intake, feed utilization efficiency, and immune capacity, thereby inhibiting growth [40]. In the present study, water quality was maintained by daily regular water exchange to support normal shrimp growth. During the 8-week feeding trial, dissolved oxygen was kept above 5.0 mg/L, ammonia-N ≤ 0.1 mg/L, and nitrite-N ≤ 0.01 mg/L. Accordingly, water quality and dissolved oxygen were not the primary limiting factors in this experiment, and the chronic stress was mainly attributed to the stocking density (crowding). Under the high-density rearing conditions used in this trial, the dietary VA supplementation level of 12,000 IU/kg (analyzed value: 12,647 IU/kg diet) yielded optimal growth performance for *L. vannamei*. Based on the second-order regression analysis of weight gain rate, the dietary VA level for maximum weight gain was estimated to be 22,800 IU/kg diet. Collectively, the optimal VA requirement determined under the present high-density culture condition is higher than those reported under conventional low-density conditions. It should be noted that the interval between the last two VA concentration gradients in this experiment was relatively large. As a result, the concentration predicted by the model exceeded the concentration corresponding to the observed optimal growth performance in the trial, which introduces uncertainty and warrants further experimental validation. Interestingly, shrimp growth declined in the final concentration gradient of this experiment. This suggests that exceeding the VA concentration that yields maximum growth may immediately inhibit shrimp growth.

The whole-body crude ash content effectively reflects the mineral deposition level of shrimp, which is predominantly composed of calcium and phosphate mineral salts derived from the exoskeleton. In the present study, *L. vannamei* in A3 showed a significantly higher whole-body crude ash content compared with the non-supplemented group A0 and low-dose VA group A1. Appropriate dietary VA might contribute to the elevated whole-body crude ash content in *L. vannamei*, possibly via synergistic regulation of calcium–phosphorus metabolism with vitamin D, improved intestinal mineral uptake, and promotion of exoskeleton calcification [18]. The active metabolite of VA, retinoic acid, could modulate calcium and phosphorus metabolism as well as biomineralization through the retinoic acid receptor (RAR)/retinoid X receptor (RXR) heterodimer pathway. Given that RAR and VDR share the RXR co-receptor, optimal VA levels may synergize with vitamin D to favor calcium and phosphorus absorption and exoskeleton mineral deposition [41,42]. Furthermore, VA is likely involved in maintaining the intestinal epithelial barrier and mucosal integrity, such as tight-junction function, which would support efficient absorption of calcium, phosphorus, and magnesium in shrimp [11].

### 4.2. Hepatopancreas Histomorphology

VA contributes to the regulation of epithelial cell proliferation and differentiation, supports tissue repair, and preserves epithelial barrier integrity [11]. In the present study, H&E staining of the hepatopancreas (Figure 3) showed that VA deficiency (A0 group) caused deformation of the hepatopancreatic tubule structure, irregular epithelial cell arrangement with detachment, and vacuolization in some cells, indicating that VA deficiency under high-density culture conditions leads to structural damage of hepatopancreas tissue. As dietary VA increased to 12,000 IU/kg (A3 group), the hepatopancreatic tubule structure gradually normalized, tubules were more tightly arranged, and epithelial cells were intact, demonstrating that an appropriate VA level can effectively maintain the normal histomorphology and structural function of the hepatopancreas in *L. vannamei* under high-density culture conditions. Analogous observations have been reported in other aquatic species: grass carp (*Ctenopharyngodon idella*) subjected to acute hypoxia stress exhibited hepatocyte swelling, nuclear displacement, and even disappearance; feeding diets containing 1614–2786 IU/kg VA significantly alleviated hypoxia-induced liver tissue damage and maintained the ultrastructural integrity of cell membranes and organelles such as mitochondria and endoplasmic reticulum [13]. Chinese mitten crab (*Eriocheir sinensis*) fed diets containing 40,000–80,000 IU/kg VA effectively repaired hepatopancreas damage caused by polychlorinated biphenyl exposure, restoring the lipid droplet content and regular tissue arrangement in the hepatopancreatic tubules [43]. These results are largely consistent with our findings, further demonstrating that VA helps maintain the hepatopancreas tissue structure and enhances tissue repair capacity in crustaceans. Nevertheless, compared with the A3 group, a disorganized epithelial arrangement was observed in partial hepatopancreatic tubules of the A4 group. These observations suggest that excessive dietary vitamin A and its subsequent accumulation may aggravate the metabolic burden of *L. vannamei* and could interfere with hepatopancreatic tissue homeostasis.

### 4.3. Lipid Metabolism

The industrialized high-density aquaculture model significantly enhances basal and maintenance metabolism in shrimp, requiring greater energy expenditure to maintain osmotic balance, antioxidant defense, and immune responses; consequently, lipid oxidation becomes an important energy-supplying pathway [44,45]. Previous studies have shown that high-density culture significantly alters the lipid metabolism pattern of *L. vannamei*, promoting lipid mobilization and increasing the consumption of fat-soluble nutrients, so that the demand for fat-soluble VA may be higher than that under conventional culture conditions [46].

In the current trial, lipase activity rose significantly in the A2 and A3 groups, indicating that appropriate VA enhanced the lipid digestive capacity and provided more substrates for subsequent fatty acid oxidation. With increasing VA levels, serum TG, TCHO, and LDL-C contents gradually decreased, whereas HDL-C significantly increased. Concurrently, the expression of fatty acid β-oxidation-related genes *cpt-1* and *aco* was significantly upregulated, while the expression of lipogenic genes *acc* and *fas* was significantly downregulated, with the most pronounced changes observed in the A3 group. CPT-1 serves as the rate-limiting enzyme governing mitochondrial fatty acid entry for β-oxidation, and ACO is the key enzyme for peroxisomal β-oxidation; their upregulated expression generally indicates enhanced fatty acid oxidation capacity [47]. Conversely, ACC and FAS are key rate-limiting enzymes for de novo fatty acid synthesis, and their downregulated expression indicates suppression of lipogenesis [48]. Therefore, the present results demonstrate that the promoting effect of VA on lipid metabolism operates both at the digestive stage and at the stages of lipid mobilization and catabolic utilization; appropriate VA levels can promote fatty acid oxidation and improve lipid utilization efficiency, thereby meeting the increased energy demands under high-density culture conditions. This likely explains how suitable VA improved the growth and health status of *L. vannamei* under these high-density culture conditions.

Nevertheless, excess VA can induce adipocyte differentiation, promote lipid deposition, and interfere with hepatopancreatic tissue homeostasis, eventually leading to growth retardation [49,50,51]. When dietary VA supplementation was further increased to 24,000 IU/kg, the whole-body crude lipid content of shrimp increased significantly. Compared with group A3, the transcript levels of *cpt-1* and *aco* were downregulated, while *acc* was upregulated, suggesting that high VA levels could perturb lipid metabolic homeostasis. As a fat-soluble vitamin, intestinal absorption of VA is not tightly controlled by negative-feedback regulation. Excess VA is primarily stored in the hepatopancreas, and its high accumulation may trigger functional alterations at the cellular level [44]. Elevated dietary VA can alter the hepatopancreatic tissue status in crustaceans and perturb both lipid homeostasis and redox balance [13,43]. At the conserved cellular level, excessive retinol can induce mitochondrial permeability transition (MPT) and cytochrome c release, boost reactive oxygen species (ROS) production, cause membrane lipid peroxidation and DNA oxidative modification, and activate the caspase cascade via the mitochondrial pathway to facilitate apoptosis [13,40,43]. A previous study on red swamp crayfish (*Procambarus clarkii*) reported that high-dose VA (243,000 IU/kg) also retarded growth and reshaped the expression profile of lipid metabolism-related genes, which is consistent with our observations in the high VA groups [44]. Collectively, excess VA may perturb hepatopancreatic homeostasis through the cascade of “oxidative stress–mitochondrial dysfunction–apoptosis/altered tissue status”. This could underlie the observed growth restriction and reshaped lipid metabolism under high dietary VA conditions.

### 4.4. Protein Synthesis

Protein deposition is a fundamental determinant of growth performance in crustaceans, and its efficiency is primarily co-regulated by the nutrient absorption capacity and protein synthesis signaling pathways. High-density culture conditions increase energy expenditure, reduce feed intake and digestive capacity, and suppress protein anabolic metabolism, ultimately leading to growth restriction [40]. In the present study, trypsin activity was significantly elevated in the A2 and A3 groups; enhanced digestive enzyme activity allowed more amino acids to enter the body, providing substrates for TOR signaling pathway-mediated protein synthesis and thereby contributing to protein deposition and organismal growth. The target of rapamycin (TOR) signaling pathway is closely associated with the growth rate and protein deposition in *L. vannamei* [52,53]; it is the core nutrient-sensing pathway regulating protein synthesis in eukaryotes, in which TOR promotes mRNA translation initiation and ribosomal protein synthesis by phosphorylating S6K and 4E-BP, thereby enhancing protein synthesis efficiency [54]. The present study found that with increasing dietary VA levels, the expression of *tor*, *s6k*, and *4e-bp* was significantly upregulated, peaking in the A3 group (12,000 IU/kg), while whole-body crude protein content and growth performance were both significantly improved, indicating that appropriate VA can promote protein synthesis and deposition in *L. vannamei* under high-density culture conditions.

At the molecular level, this regulatory effect may occur at the level of intracellular amino acid sensing. Dietary VA is converted to all-trans retinoic acid (ATRA), which may promote the expression of amino acid transporters and thereby expand intracellular amino acid pools available for mammalian target of rapamycin complex 1 (mTORC1) activation [55,56]. For example, leucine subsequently activates the Sestrin2-GATOR2-Rag cascade, which recruits and activates mTORC1 at the lysosomal membrane and, in turn, phosphorylates S6K and 4E-BP to drive protein translation [53]. The Sestrin2-Rag-TOR axis is evolutionarily conserved in *L. vannamei*; more leucine downregulates sesn gene expression in the hepatopancreas while upregulating the expression of genes including *tor*, *akt*, *slc3a2*, *s6k*, and *eif4e2* [52,54,57].

### 4.5. Antioxidant Capacity

Prolonged stress disrupts the oxidative–antioxidant balance in shrimp, particularly causing significant damage to the hepatopancreas, which serves as the central organ of metabolism and immunity [58]. Therefore, enhancing antioxidant capacity is an important strategy for alleviating culture-related stress such as high stocking density and water quality deterioration. VA and its metabolite retinoic acid not only scavenge free radicals and inhibit lipid peroxidation but also maintain cell membrane stability and reduce oxidative stress-induced tissue damage [11]. Numerous studies have shown that appropriate VA supplementation can enhance antioxidant enzyme activities and reduce MDA accumulation in fish and crustaceans, thereby improving the organism’s adaptability to environmental stress [37,59,60]. In the present study, PO, GSH-Px, and SOD activities generally increased with increasing dietary VA levels, whereas the MDA content significantly decreased, with the optimal antioxidant status observed in the A3 group. These results indicate that appropriate VA can effectively enhance the antioxidant defense capacity of *L. vannamei* and mitigate lipid peroxidation damage. Gene expression results were largely consistent with the enzyme activity changes; *sod*, *po*, and *gshpx* expression were significantly upregulated with increasing VA levels, indicating that VA not only enhances antioxidant enzyme activities but also regulates the antioxidant defense system at the transcriptional level.

### 4.6. Apoptosis Alleviation and Immune Pathway Activation

Unlike vertebrates, *L. vannamei* lacks a typical adaptive immune system; its defense relies mainly on the innate immune system composed of hemocytes, hepatopancreas, and intestine, which encompasses immune effectors such as antimicrobial peptides, lysozyme, and apoptosis-regulatory pathways [61,62]. Nevertheless, shrimp are not entirely devoid of immune “memory”. Recent studies have confirmed that invertebrates exhibit immune priming, whereby an initial encounter with a pathogen or antigen produces an enhanced protective response lasting from days to weeks, and this phenomenon has been demonstrated repeatedly in *L. vannamei* [63,64]. These innate immune effectors form a critical line of defense against pathogen infection, and their expression levels directly reflect the host’s immune status. Hence, their immune defense is highly sensitive to the nutritional status and environmental conditions and is therefore readily compromised by stress factors in intensive culture.

The industrialized high-density aquaculture model, characterized by high stocking density, high feeding rates, and limited water exchange, results in a predisposition to the accumulation of ammonia nitrogen, nitrite, and organic matter and increases the risk of pathogen transmission [36]. High-density stress can also cause oxidative damage and immune dysfunction, suppressing immune-related signaling pathways and thereby reducing disease resistance [65]. LZM exerts direct bactericidal effects by hydrolyzing peptidoglycan in bacterial cell walls, while Crustins, as crustacean-specific antimicrobial peptides, play important roles in inhibiting Gram-positive bacteria and maintaining intestinal microbial homeostasis [66]. The present findings indicate that appropriate VA supplementation significantly upregulated *lzm* and *crustins* expression, with the A2 and A3 groups showing higher levels, indicating that appropriate VA can enhance the synthesis of innate immune effectors in *L. vannamei* and improve defense against potential pathogens. Concurrently, *hsp70* and *aif* expression decreased with increasing VA levels, reaching their lowest levels in the A3 group. HSP70 is an important molecular chaperone during the cellular stress response; its elevated expression typically reflects strong environmental stress. AIF is a key regulator of the mitochondria-dependent apoptosis pathway, and its increased expression is often associated with greater cell damage and apoptosis [67]. Combined with the hepatopancreas histomorphology results (Figure 3), these findings indicate that increasing VA levels reduced tissue stress and alleviated apoptotic pressure, thereby mitigating cellular stress responses and tissue damage under high-density culture conditions, maintaining cellular homeostasis, and providing favorable conditions for normal immune function.

It is noteworthy that significant differences in apoptosis-related markers were observed between the A3 and A4 groups. In the A3 group (analyzed dietary VA: 12,647 IU/kg), the hepatopancreas retained an intact tissue architecture, with low transcript levels of *aif* and *hsp70*. By comparison, the shrimp in the A4 group receiving higher dietary VA (24,758 IU/kg) exhibited altered hepatopancreatic morphology together with upregulated expression of *hsp70* and *aif* (Figure 3). These observations suggest that a critical shift in physiological response may occur within the dose range between A3 and A4, which is presumably linked to a dose-dependent physiological threshold of intracellular retinoic acid. Previous studies have demonstrated that moderate-to-low retinoic acid can exert anti-apoptotic effects by upregulating B-cell lymphoma 2 (Bcl-2) and repressing Bcl-2-associated X protein (Bax) and caspase-3 [68]. Once intracellular retinoic acid exceeds this physiological threshold, cellular responses may shift toward pro-apoptotic profiles, characterized by downregulated Bcl-2 and activated caspase-3 [69]. Elevated retinoic acid can further modulate the p14-MDM2-p53 cascade and RAR-γ receptor signaling, triggering downstream caspase-dependent mitochondrial apoptosis, including Bax, p53 upregulated modulator of apoptosis (PUMA), BH3-interacting domain death agonist (Bid), caspase-9, caspase-8, caspase-3, and poly (ADP-ribose) polymerase (PARP) [69,70]. The *rxr* gene was highly expressed in the hepatopancreas of *L. vannamei*. Its encoded protein forms heterodimers with other nuclear receptors, providing a molecular foundation for retinoid signal transduction [71,72]. Accordingly, the higher dietary VA in the A4 group might drive hepatopancreatic retinoic acid past this physiological threshold, consequently upregulating AIF-dependent mitochondrial apoptotic signaling as well as the HSP70-mediated chaperone stress response. This interpretation was consistent with the elevated *hsp70/aif* transcripts and morphological changes observed in group A4. Nevertheless, this mechanistic inference was based solely on transcript data; further validation, including tissue retinoic acid quantification, measurement of Bcl-2/Bax protein abundance, and caspase activity assays, is still required.

The synthesis of innate immune effectors is regulated by multiple immune signaling pathways, among which the Toll and immune deficiency (IMD) pathways are the two most important immune regulatory pathways for crustacean defense against pathogen infection [62]. Previous studies have shown that VA, through its active metabolite retinoic acid binding to RAR/RXR receptors, can regulate the transcription of downstream immune-related genes and participate in immune function regulation [73]. In the present study, the transcript levels of *rxr* in groups A2 and A3 were significantly higher than those in group A0, suggesting that adequate VA may participate in retinoic acid signal transduction. Significant upregulation of *tlr4*, *myd88*, and *nf-κB* was observed in group A3. As a key pattern-recognition receptor, TLR4 recognizes pathogen-associated molecular patterns and can mediate the NF-κB signaling cascade via MyD88 to induce the expression of multiple immune effector molecules [74]. Meanwhile, the expression of *imd* and *relish* was also markedly increased in groups A2 and A3. The IMD-Relish pathway represents a vital signaling cascade for defense against bacterial infection in crustaceans, and its activation promotes the synthesis of antimicrobial peptides and related immune factors [75]. Taken together with the concurrent upregulation of *lzm* and *crustins* in our study, it is hypothesized that elevated transcription of genes associated with the TLR4-MyD88-NF-κB and IMD-Relish cascades under adequate VA may contribute to the synthesis and release of immune effector molecules, which could improve the non-specific immune status of *L. vannamei* under high-density culture conditions.

## 5. Conclusions

Under intensive indoor high-density culture conditions, appropriate dietary VA supplementation contributes to improved growth performance, nutritional metabolic status, antioxidant defense, and innate immune-related responses in *L. vannamei* and can partially mitigate hepatopancreatic morphological alterations and oxidative stress induced by a high stocking density. Within the dose gradient tested in the present study, further elevation of dietary VA did not produce continuous improvement in these parameters, and reversed trends were observed for several indicators. Under the high-density farming system in the present study, the 12,647 IU/kg dietary VA could improve *L. vannamei* growth. Based on the weight gain rate, the feed VA concentration required for shrimp to achieve maximum growth at this density is 22,800 IU/kg. This value is higher than that reported under conventional rearing conditions, and these data suggested that long-term crowding stress and multiple stressors derived from high-density environments may modify the physiological requirements for antioxidant-related nutrients. However, the 22,800 IU/kg value, estimated via second-order regression analysis based on weight gain, is a model-predicted result subject to uncertainty, and further trials with more refined concentration gradients are required for verification.

The present findings provide practical economic references for commercial *L. vannamei* farming. Feed constitutes the highest variable cost in shrimp aquaculture. Extrapolated from the initial weight, final weight, and weight gain rate (WGR) in this trial, individual weight gain in the optimal VA group (A3) increased by 19.25% relative to the VA-deficient group (A0) under identical initial body weight. Theoretical calculation based on the stocking density of the experimental tanks shows that with equal survival rate, the optimal VA treatment yields an extra shrimp production of approximately 19.6 kg at a stocking of 20,000 shrimp, corresponding to a volumetric yield gain of 0.7 kg/m^3^. These estimations are derived from indoor tank data; actual pond-scale production is further influenced by survival rate, disease pressure, and farm-management practices.

In addition, improved growth performance under adequate VA reduces feed consumption per unit output, together with nitrogen-phosphorus excretion and carbon footprint. Meanwhile, optimal dietary VA modulates antioxidant-signaling pathways, offering theoretical support for precision nutrition strategies to alleviate high density-related stress, mitigate disease-caused losses, and cut antibiotic usage. This is in line with aquaculture policies for antibiotic reduction and antimicrobial-resistance containment. Furthermore, for commercial feed manufacturers, we recommend formulating diets with a VA overage of 15–20% above the target analyzed concentration to account for losses during extrusion, drying, and storage. Feed should be stored at ≤25 °C in light-proof packaging, and VA retention should be verified by post-production analysis within 30 days of manufacture.

## 6. Limitations of the Present Study

Several limitations exist in the present study. Firstly, only a single stocking density (720 shrimps per cubic meter) was adopted in the experiment referenced in the practical. Secondly, the results for the current study were only carried out on juvenile shrimp with the basal feed formula. The VA requirement of shrimp varies at different growth stages, and the interaction between various nutrients can also affect the VA demand to a certain extent. Thirdly, accordingly, the dietary VA concentration for the maximum weight-gain rate observed in this trial deviated from the value derived from the quadratic regression model; we underestimated the body’s requirement for vitamin A under high-density rearing conditions. Fortunately, a dose that might be toxic has been considered. Future work shall adopt finer VA dosage intervals between 18,000 and 24,000 IU/kg on the basis of the current results. Finally, the molecular-level results of this study were solely based on the detection of mRNA expression, which cannot directly verify the activation of related signaling pathways, protein phosphorylation status, or functional phenotypes. Thus, the mechanisms through which VA regulates shrimp health remain to be further explored.

## Figures and Tables

**Figure 1:**
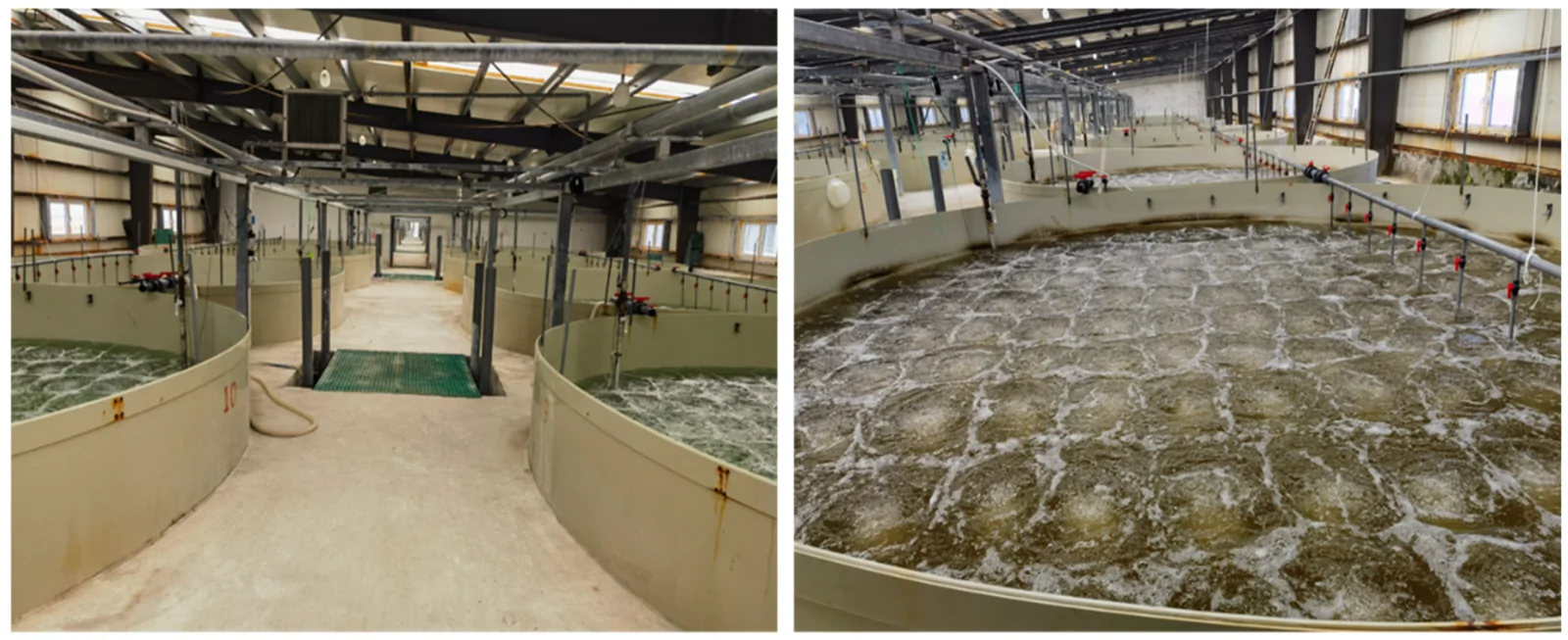
Industrial high-density shrimp rearing facility used in the feeding trial.

**Figure 2:**
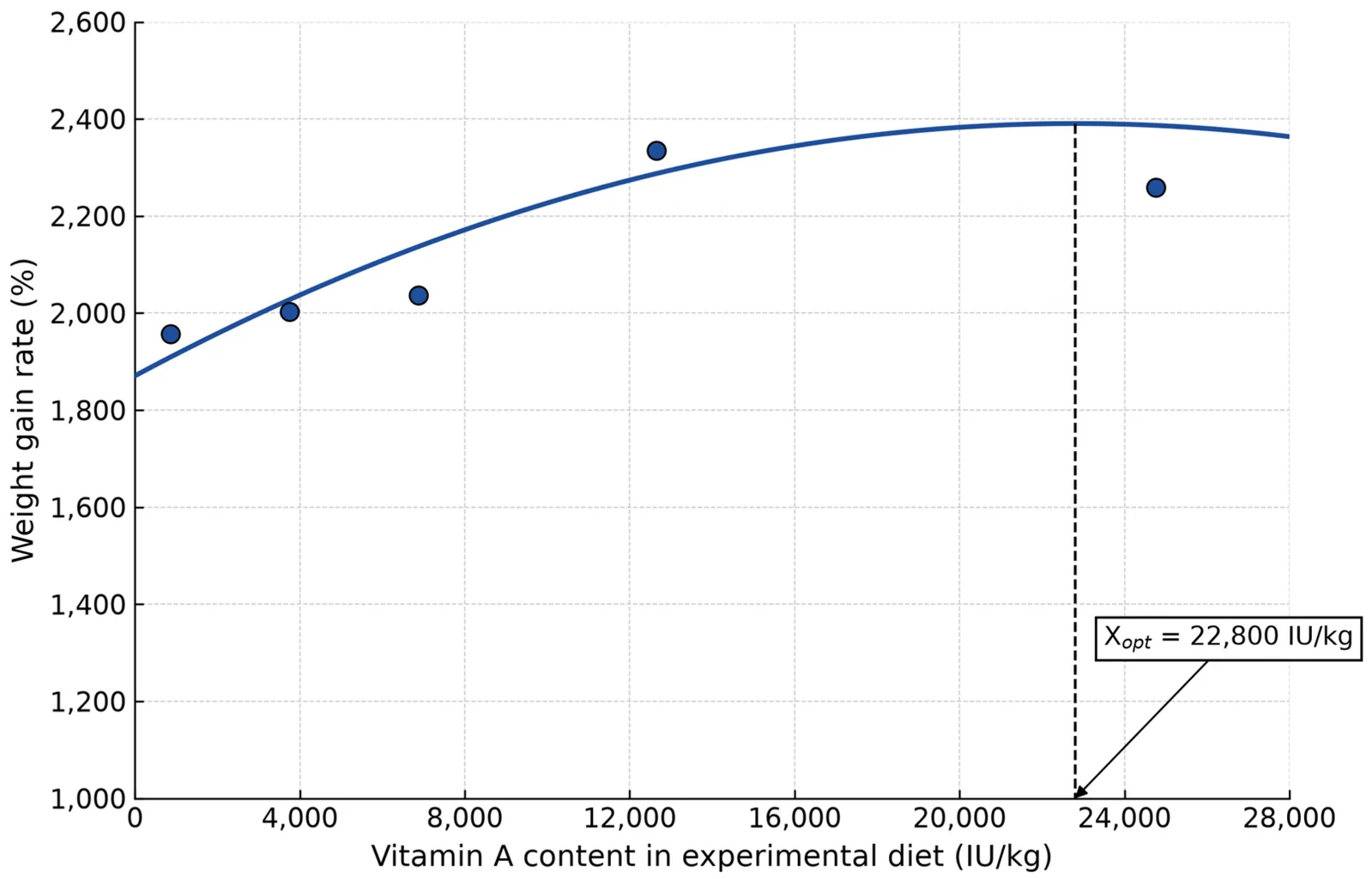
Quadratic regression analysis of weight gain rate (WGR) of *L. vannamei* as a function of dietary VA level.

**Figure 3:**
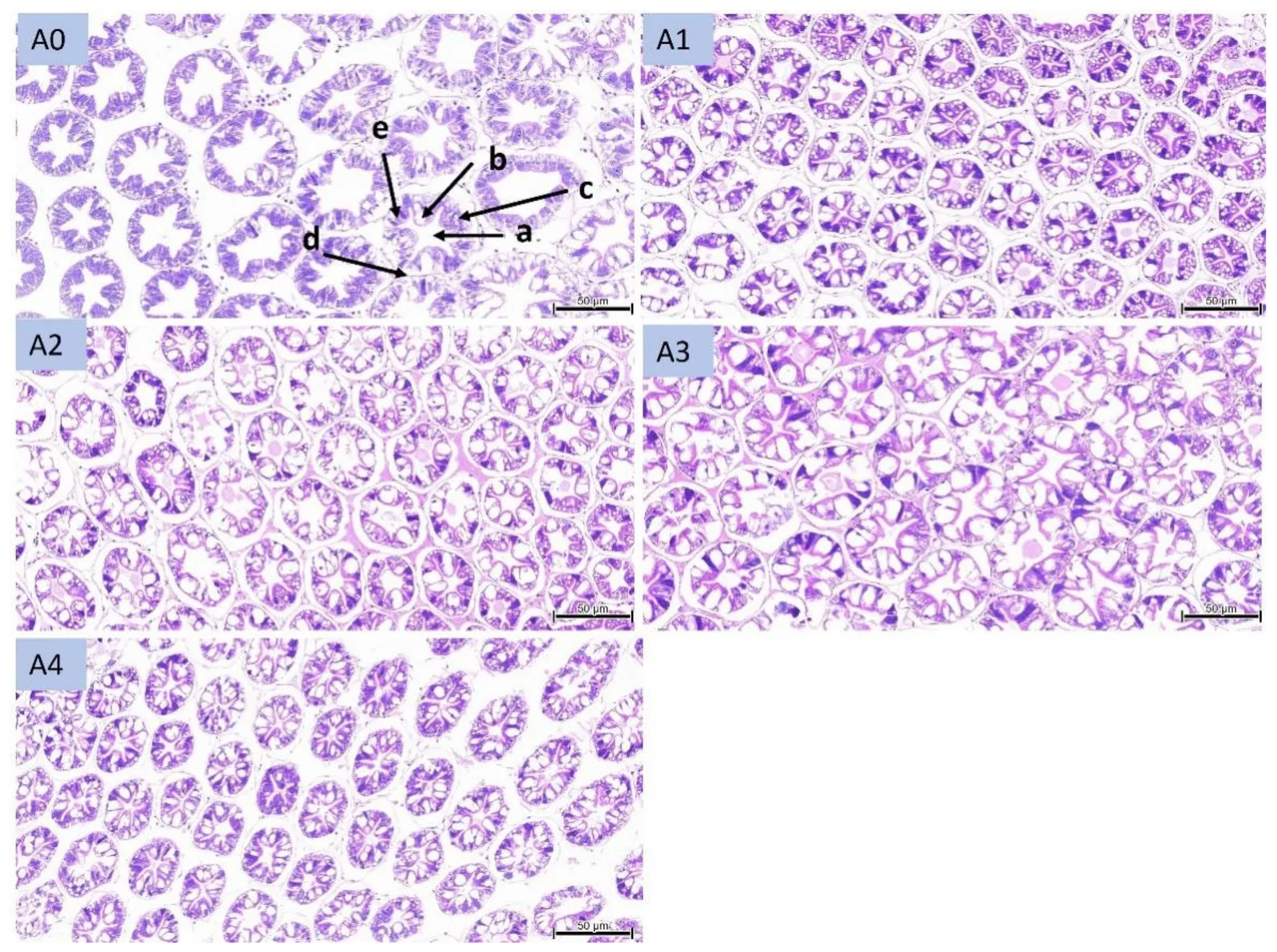
Effects of dietary VA levels on the hepatopancreas histological morphology of *L. vannamei* (magnification: 400×; scale bar = 50 μm). (a) Hepatopancreatic tubules; (b) B cell (digestive cells); (c) R cell (absorptive cells); (d) basement membrane; (e) E cell (embryonic cells).

**Figure 4:**
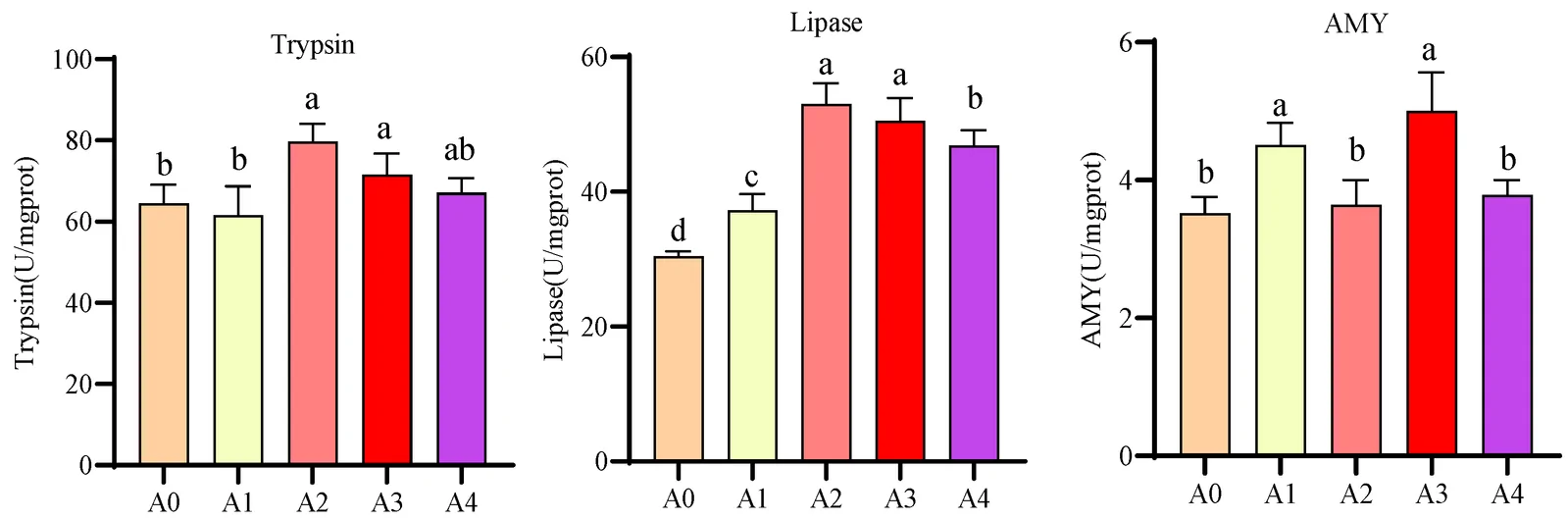
Effects on digestive enzyme activities in the hepatopancreas of shrimp. Values with different superscript letters indicate significant differences among treatments (*p* < 0.05).

**Figure 5:**
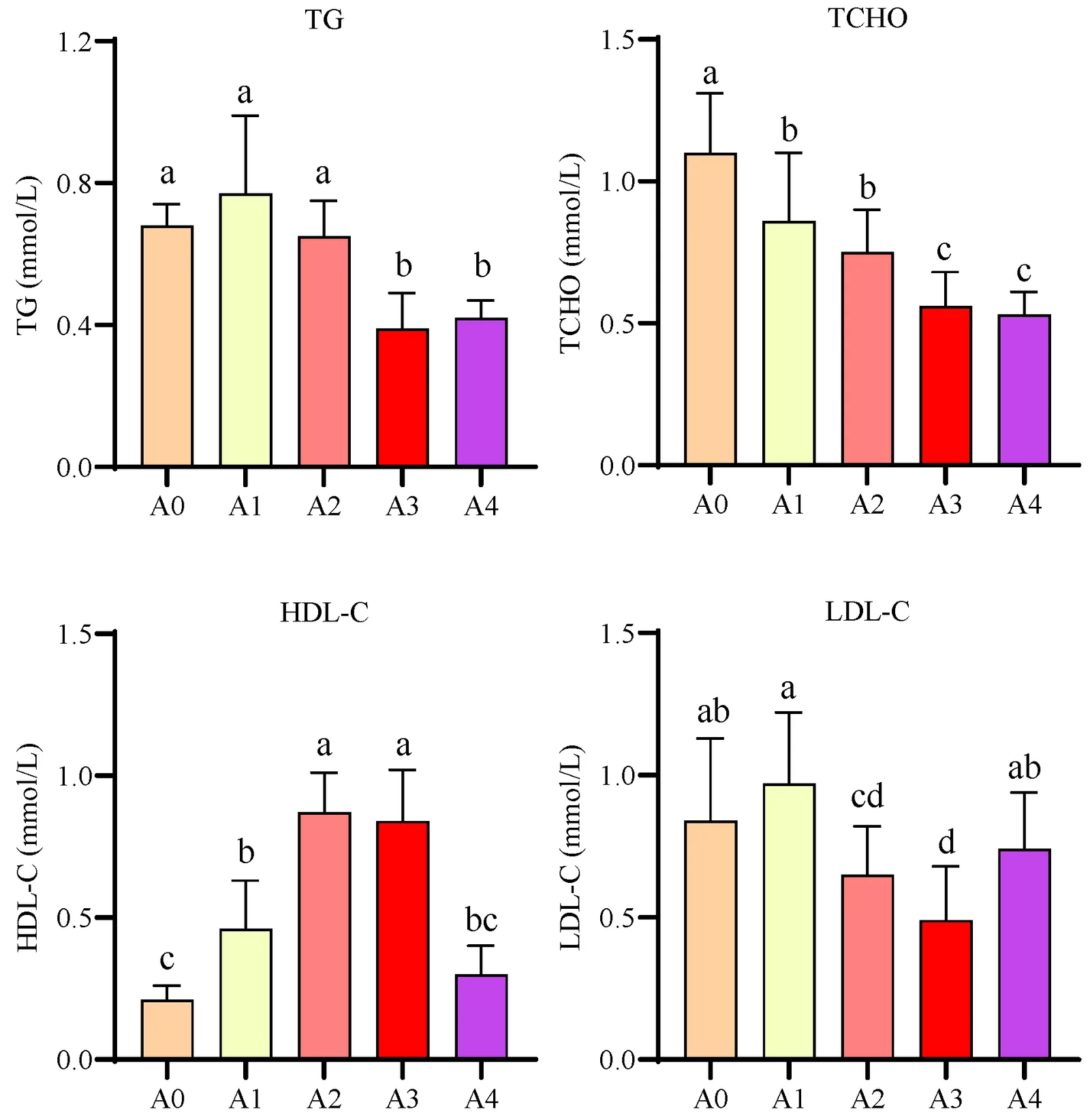
Effects of dietary VA levels on serum biochemical parameters of *L. vannamei*. Values with different superscript letters indicate significant differences among treatments (*p* < 0.05).

**Figure 6:**
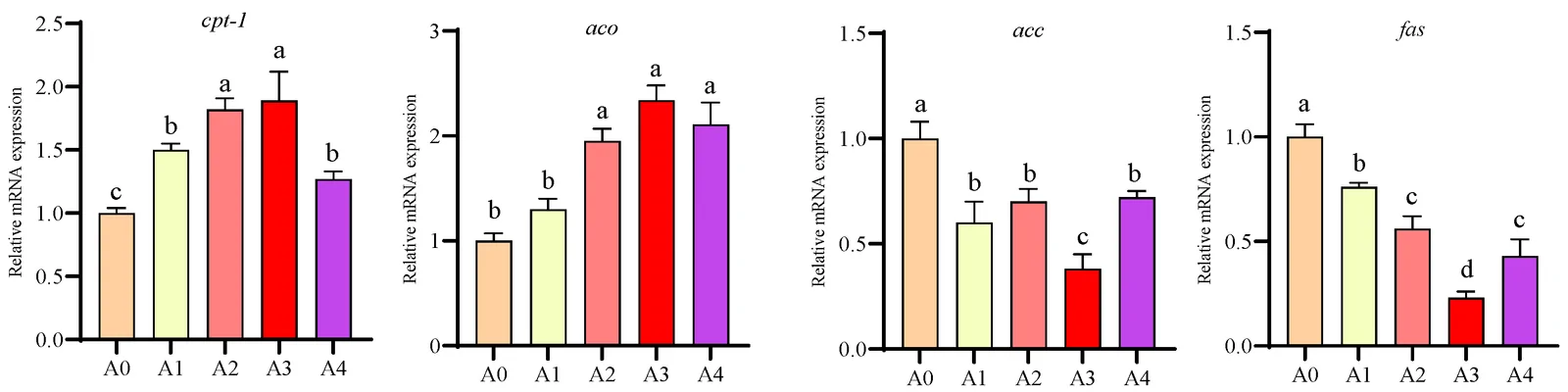
Effects of dietary VA levels on the expression of lipid metabolism-related genes in *L. vannamei*. Values with different superscript letters indicate significant differences among treatments (*p* < 0.05).

**Figure 7:**
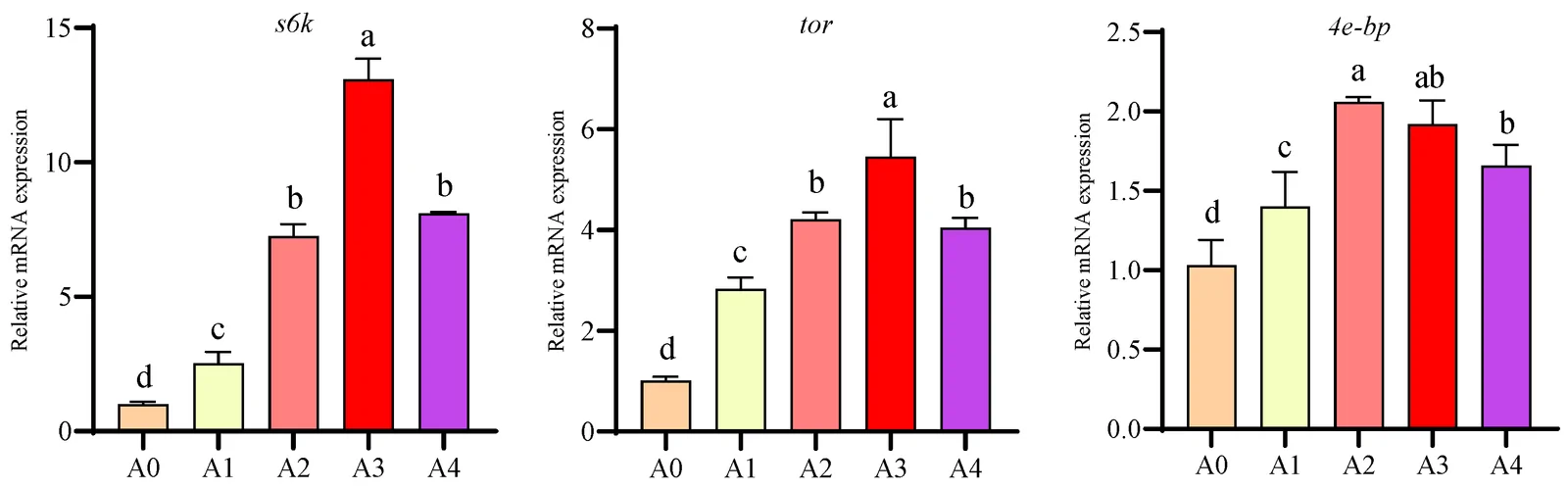
Effects of dietary VA on the expression of protein synthesis-related genes in *L. vannamei*. Values with different superscript letters indicate significant differences among treatments (*p* < 0.05).

**Figure 8:**
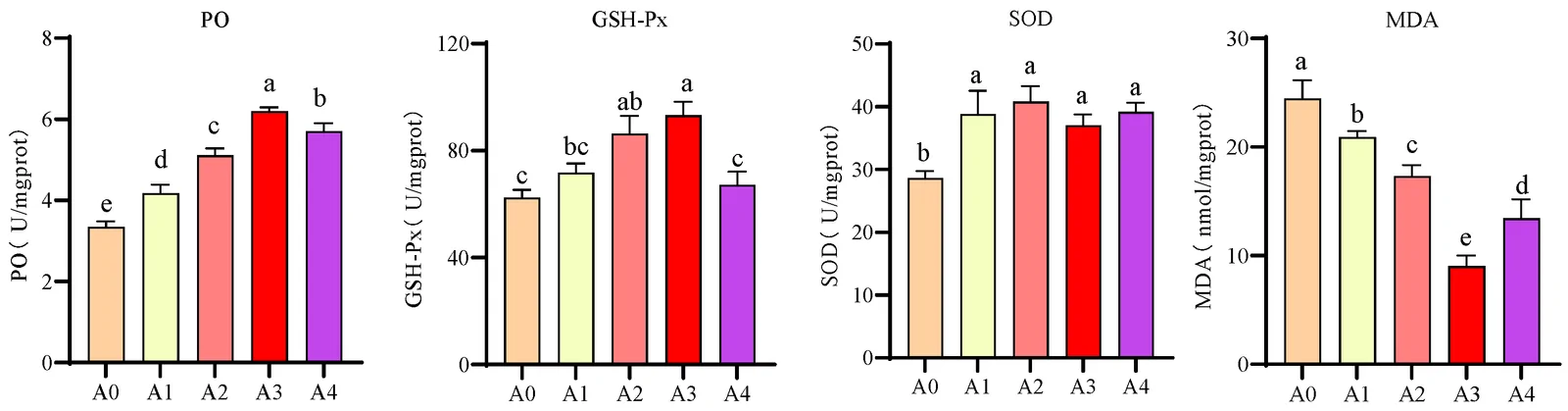
Effects of dietary VA on hepatopancreatic antioxidant enzyme activities in *L. vannamei*. Values with different superscript letters indicate significant differences among treatments (*p* < 0.05).

**Figure 9:**
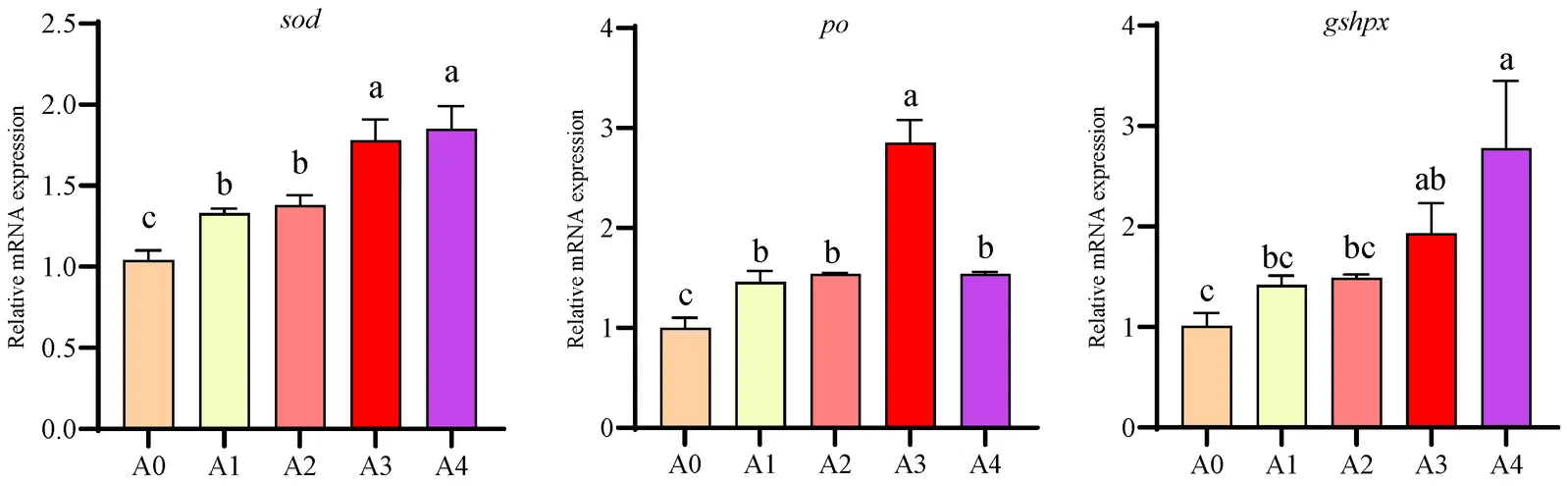
Effects of dietary VA on the expression of antioxidant-related genes in *L. vannamei*. Values with different superscript letters indicate significant differences among treatments (*p* < 0.05).

**Figure 10:**
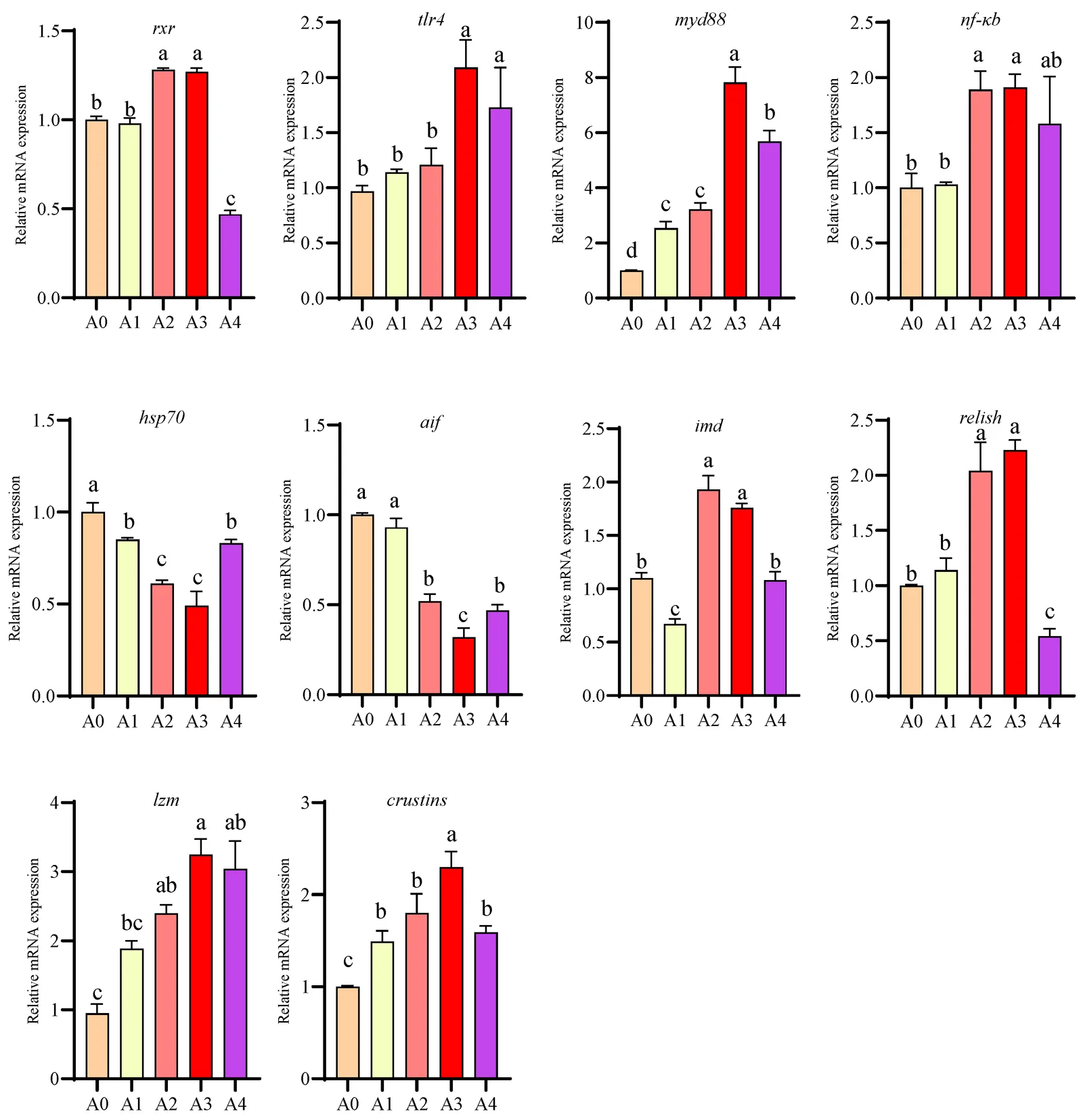
Effects of dietary VA on the expression of immune regulatory genes in *L. vannamei*. Values with different superscript letters indicate significant differences among treatments (*p* < 0.05).

**Figure 11:**
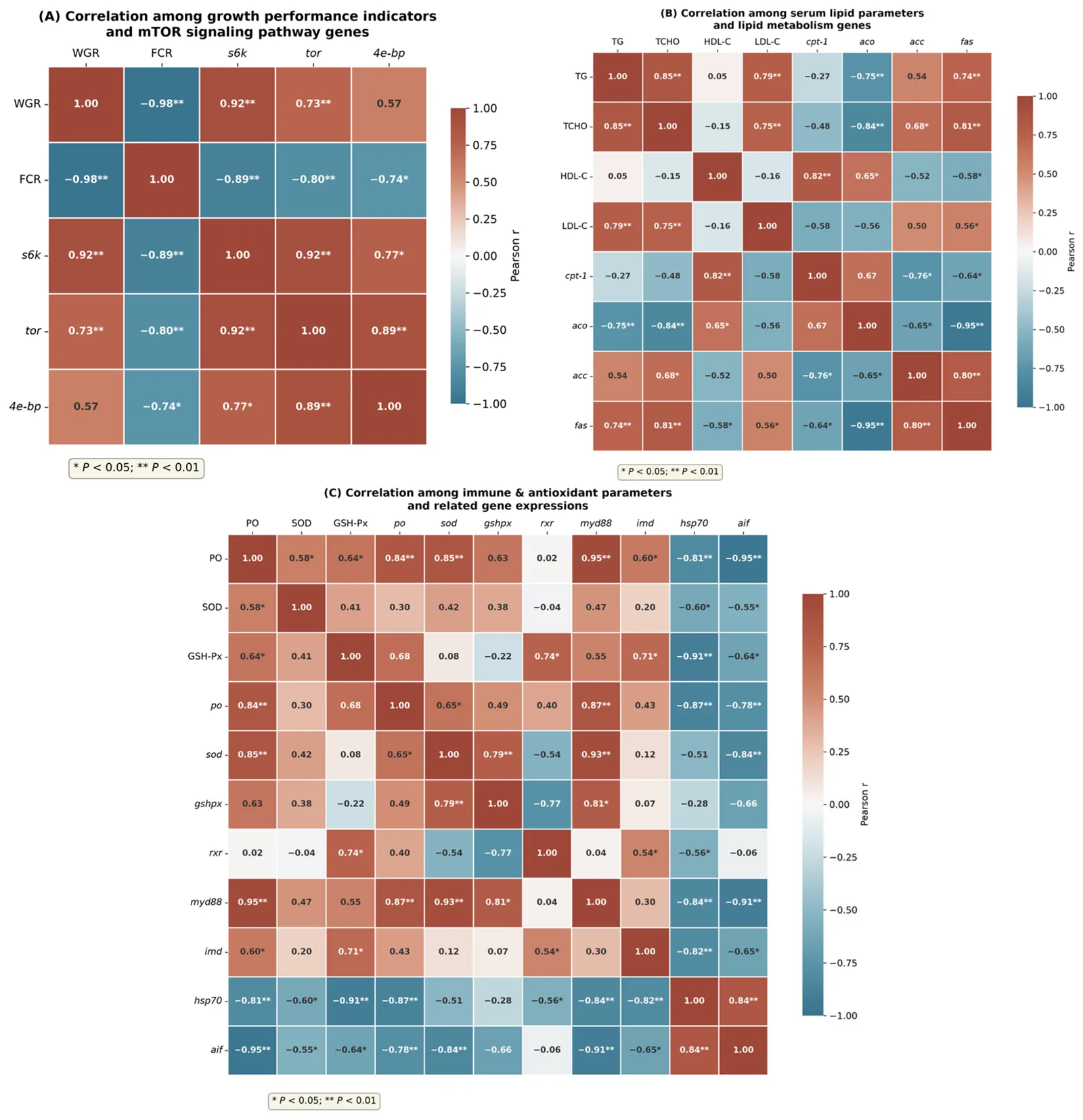
Pearson correlation heatmap of growth performance, lipid metabolism, immune-antioxidant indices, and their related gene expression. (**A**) Growth performance and mTOR signaling pathway genes; (**B**) serum lipid parameters and lipid-metabolism-related genes; (**C**) immune-antioxidant parameters and immune-antioxidant-related genes. Red indicates a positive correlation, and blue indicates a negative correlation. * *p* < 0.05, ** *p* < 0.01.

**Table 1:** Composition and nutrient levels of foundational diets (%).

Ingredients	Contents
Fish meal	30.0
Poultry by-product meal	5.0
Soybean meal	15.0
Peanut meal	6.0
Prawn shell powder	5.0
Wheat flour	23.0
Spray-dried chicken hemoglobin powder	2.5
Fish paste	7.0
Soybean lecithin oil	3.0
Calcium dihydrogen phosphate	2.0
Choline chloride	0.4
Vitamin premix ^a^	0.1
Mineral premix ^b^	1.0
Total	100.0
Proximate chemical composition (dry-matter basis %)	
Moisture	10.08
Crude protein	45.20
Crude lipid	7.12
Ash	9.49

^a^. Vitamin premix provided the following nutrients per kg of premix: thiamin, 0.5 g; riboflavin, 0.7 g; pyridoxine hydrochloric, 0.6 g; vitamin B12, 0.1 mg; vitamin K3, 10 mg; inositol, 8 g; pantothenic acid, 60 mg; nicotinic acid, 200 mg; folic acid, 0.15 g; biotin, 1.2 mg; vitamin D3, 5 mg; α-tocopherol, 5 g; ethoxyquinoline, 150 mg; microcrystalline cellulose, 1511.7 mg. ^b^. Mineral premix provided the following nutrients per kg of premix: MgSO_4_·H_2_O, 4000 mg; MnSO_4_·H_2_O, 50 mg; KI, 100 mg; CoCl_2_(1%), 100 mg; CuSO_4_·5H_2_O, 20 mg; FeSO_4_·H_2_O, 260 mg; ZnSO_4_·H_2_O, 150 mg; Na_2_SeO_3_(1%), 50 mg.

**Table 2:** Primer used for real-time PCR.

Genes	Accession Number	Primers	Amplicon Size (bp)	Amplification Efficiency (%)
*LvEF1α*	GU136229.1	F: TATGCTCCTTTTGGACGTTTTGC	100	92.3
		R: CCTTTTCTGCGGCCTTGGTAG		
*propo*	XM_070133708.1	F: TACATGCACCAGCAAATTATCG	134	*95*
		R: AGTTTGGGGAAGTAGCCGTC		
*sod*	XM_027370407.2	F: GCAATGAATGCCCTTCTACC	144	94
		R: CAGAGCCTTTCACTCCAACG		
*gshpx*	XM_070144014.1	F: TTTATGTCAGATCCCAAAGTGCATCA	128	93
		R: CAGCAAGTTTGCGATTTCATCTTTA		
*fas*	HM595630.1	F: CGTGACACCCCTTCCTCAC	134	99
		R: GAGAGTGTGAGGAACATAGACCA		
*acc*	XM_070132326.1	F: CGGCAGACAACATCCATACCACAG	150	100
		R: GCAACCAGCGAGAGCAGTAACC		
*cpt-1*	XM_027361886.1	F: AGATTGCCTCTGCCTGC	122	98.4
		R: AAAGCAGCGTGGGTGAC		
*aco*	XM_070118862.1	F: CTTCCGCACCAACCCACT	145	98.6
		R: GCTTCAGGACTGTTCCACCA		
*tor*	XP_027228160.1	F: TGCCAACGGGTGGTAGA	141	99
		R: GGGTGTTTGTGGACGGA		
*s6k*	XM_027380602.2	F: GCAAGAGGAAGACGCCATA	122	97
		R: CCGCCCTTGCCCAAAACCT		
*4e-bp*	XM_027367939.2	F: ATGTCTGCTTCGCCCGTCGCTCGCC	150	98.4
		R: GGTTCTTGGGTGGGCTCTT		
*tlr4*	XM_070135261.1	F: CGCTGTCCGCTCTACCA	98	99.3
		R: CACCGTCTTGTTCCTATCGTC		
*myd88*	JX073568.1	F: TAGATCAGCGTCGTCGTA	93	99.7
		R: GAGGGAGTGGCAGAAAC		
*nf-kB*	XM_027371626.2	F: TGTGGTTTACGAGAGGCT	91	98
		R: GTTCCAACAAAGGAGGTG		
*imd*	FJ592176.1	F: CGGCTCTGCGGTTCACAT	108	98
		R: CCTCGACCTTGTCTCGTTCCT		
*relish*	EF432734.1	F: CTACATTCTGCCCTTGACTCTGG	104	99.6
		R: GGCTGGCAAGTCGTTCTCG		
*hsp70*	DQ813343.1	F: AGGAGACCGCTGAGGCTTAC	104	100
		R: AGCACATTCAGACCCGAGAT		
*rxr*	XM027361142.1	F: GGACAAAGGGAGATAAAGAA	122	102
		R: CTGTTGGGTCTGAGTTGAG		
*aif*	XM_070129927.1	F: GGATGTGGTGTCCTGGATGG	134	108.5
		R: GCGTCGTCCTCCGTGATG		
*crustins*	AY488497.1	F: GGTGTTGGTGGTGGTTTCCC	145	107
		R: CAGTCGCTTGTGCCAGTTCC		
*lzm*	XM_027357011.2	F: AAGGCTATTCTGCCTGGGCT	157	104
		R: TGGAAACCCTTGGTGACAAGC		

Notes: *propo*, phenoloxidase; *sod*, superoxide dismutase; *gshpx*, glutathione peroxidase; *fas*, fatty acid synthase; *acc*, acetyl-CoA carboxylase; *cpt-1*, carnitine palmitoyltransferase 1; *aco*, acyl-CoA oxidase; *tor*, target of rapamycin; *s6k*, ribosomal protein S6 kinase; *4e-bp*, eukaryotic translation initiation factor 4E-binding protein; *tlr4*, toll-like receptor 4; *myd88*, myeloid differentiation primary response gene 88; *nf-kB*, nuclear factor kappa B; *imd*, immune deficiency; *relish*, relish nuclear factor kappa B; *hsp70*, heat shock protein 70; *rxr*, retinoid x receptor; *aif*, apoptosis inducing factor, mitochondrial 1; *crustins*, Crustins; *lzm*, lysozyme.

**Table 3:** Effects of dietary VA levels on the growth performance of *L. vannamei*.

Items	IBW (g)	FBW (g)	WGR (%)	SGR (%/d)	FCR
A0	0.26 ± 0.01	5.35 ± 0.04 ^d^	1957.69 ± 13.60 ^d^	5.93 ± 0.01 ^d^	1.18 ± 0.01 ^a^
A1	0.26 ± 0.02	5.47 ± 0.01 ^cd^	2002.56 ± 5.55 ^cd^	5.97 ± 0.01 ^cd^	1.15 ± 0.01 ^ab^
A2	0.26 ± 0.01	5.56 ± 0.08 ^c^	2037.02 ± 31.77 ^c^	6.00 ± 0.03 ^c^	1.13 ± 0.02 ^b^
A3	0.26 ± 0.02	6.33 ± 0.04 ^a^	2335.26 ± 16.36 ^a^	6.26 ± 0.01 ^a^	0.99 ± 0.01 ^d^
A4	0.26 ± 0.02	6.13 ± 0.09 ^b^	2258.97 ± 33.77 ^b^	6.20 ± 0.03 ^b^	1.02 ± 0.02 ^c^

Data are presented as mean ± SE (n = 4). Different superscripts in a row denote significant differences at *p* < 0.05; same for all subsequent tables/figures.

**Table 4:** Effects of dietary VA levels on the whole-body composition of *L. vannamei*.

Items	Moisture (%)	Crude Protein (%)	Crude Lipid (%)	Ash (%)
A0	74.87 ± 0.57 ^c^	69.89 ± 0.57 ^b^	3.79 ± 0.77 ^c^	13.29 ± 0.62 ^b^
A1	77.53 ± 0.59 ^a^	70.10 ± 0.62 ^ab^	4.76 ± 0.81 ^b^	12.75 ± 0.18 ^c^
A2	75.57 ± 0.71 ^bc^	70.47 ± 0.59 ^ab^	3.83 ± 0.32 ^c^	13.56 ± 0.1 ^ab^
A3	77.80 ± 0.59 ^a^	71.01 ± 0.70 ^a^	3.59 ± 0.28 ^c^	13.86 ± 0.04 ^a^
A4	76.13 ± 0.55 ^b^	70.65 ± 0.55 ^ab^	7.48 ± 0.40 ^a^	13.35 ± 0.12 ^b^

Note: Moisture was expressed on a wet-weight basis (%). Crude protein, crude lipid, and ash were expressed on a dry-matter basis (%). Values in the same column with different superscript letters are significantly different (*p* < 0.05).

## Data Availability

Data analyzed in the present study are included in the article.

## References

[1] Sun Y., Zhang S., Feng W., Zhang Y., Han T., Wang J. (2025). Impact of Dietary Carbohydrate Levels on Growth Performance, Feed Efficiency, and Immune Response in *Litopenaeus vannamei* Cultured in Biofloc Systems. Metabolites.

[2] Halachmi I. (2007). Biomass management in recirculating aquaculture systems using queuing networks. Aquaculture.

[3] Ray A.J., Drury T.H., Cecil A. (2017). Comparing clear-water RAS and biofloc systems: Shrimp (*Litopenaeus vannamei*) production, water quality, and biofloc nutritional contributions estimated using stable isotopes. Aquac. Eng..

[4] Suantika G., Situmorang M.L., Kurniawan J.B., Pratiwi S.A., Aditiawati P., Astuti D.I., Azizah F.F.N., Djohan Y.A., Zuhri U., Simatupang T.M. (2018). Development of a zero water discharge (ZWD)—Recirculating aquaculture system (RAS) hybrid system for super intensive white shrimp (*Litopenaeus vannamei*) culture under low salinity conditions and its industrial trial in commercial shrimp urban farming in Gresik, East Java, Indonesia. Aquac. Eng..

[5] Sookying D., Silva F.S.D., Davis D.A., Hanson T.R. (2011). Effects of stocking density on the performance of Pacific white shrimp *Litopenaeus vannamei* cultured under pond and outdoor tank conditions using a high soybean meal diet. Aquaculture.

[6] Roy L.A., Teichert-Coddington D., Beck B.H., Dahl S., James J., Guo J., Davis D.A. (2020). Evaluation of Stocking Density and Dietary Fish Meal Inclusion for Intensive Tank Production of Pacific White Shrimp *Litopenaeus vannamei* Cultured in Low-Salinity Waters of Western Alabama. N. Am. J. Aquac..

[7] Liñán-Cabello M.A., Paniagua-Michel J., Hopkins P.M. (2002). Bioactive roles of carotenoids and retinoids in crustaceans. Aquac. Nutr..

[8] Liu G., Zhu S., Liu D., Guo X., Ye Z. (2017). Effects of stocking density of the white shrimp *Litopenaeus vannamei* (Boone) on immunities, antioxidant status, and resistance against Vibrio harveyi in a biofloc system. Fish Shellfish Immunol..

[9] Li Y., Wei L., Cao J., Qiu L., Jiang X., Li P., Song Q., Zhou H., Han Q., Diao X. (2015). Oxidative stress, DNA damage and antioxidant enzyme activities in the pacific white shrimp (*Litopenaeus vannamei*) when exposed to hypoxia and reoxygenation. Chemosphere.

[10] Liang Z., Liu R., Zhao D., Wang L., Sun M., Wang M., Song L. (2016). Ammonia exposure induces oxidative stress, endoplasmic reticulum stress and apoptosis in hepatopancreas of pacific white shrimp (*Litopenaeus vannamei*). Fish Shellfish Immunol..

[11] Shastak Y., Pelletier W. (2024). Vitamin A in Fish Well-Being: Integrating Immune Strength, Antioxidant Capacity and Growth. Fishes.

[12] Lin L., Li J., Liu J., Zhuo H., Zhang Y., Zhou X., Wu G., Guo C., Zhao X. (2024). Single and combined effects of ammonia and nitrite on *Litopenaeus vannamei*: Histological, physiological and molecular responses. Aquac. Rep..

[13] Cheng H., Feng L., Wu P., Liu Y., Ma Y., Ren H., Jin X., Zhou X., Jiang W. (2025). Exploring new benefits of vitamin A: Alleviating hypoxia-induced mitochondrial stress and mitophagy in the gills of adult grass carp (*Ctenopharyngodon idella*). J. Anim. Sci. Biotechnol..

[14] Cheng H., Zhou X., Feng L., Wu P., Liu Y., Ma Y., Tang J., Wu F., Zhang L., Mi H. (2026). Acute hypoxia induces liver mitochondrial and endoplasmic reticulum stress in adult grass carp (*Ctenopharyngodon idella*): Palliative effects of vitamin A. Anim. Nutr..

[15] Kim S., Lee K.-J. Dietary Vitamin A Requirement for Pacific White Shrimp *Penaeus vannamei*. Proceedings of the Asian-Pacific Aquaculture 2024.

[16] Jiang W., Jia X., Zhang M., Qiang W., Shen X., Jiang G., Li X., Chi C., Liu H., Liu W. (2024). The effect of dietary vitamin A supplementation on growth, gonadal development, molting, immunity, and ammonia nitrogen resistance in sub-adult male Chinese mitten crab, *Eriocheir sinensis*. Aquaculture.

[17] Campeche D.F.B., Catharino R.R., Godoy H.T., Cyrino J.E.P. (2009). Vitamin A in diets for Nile tilapia. Sci. Agric..

[18] Gürbüz M., Aktaç Ş. (2022). Understanding the role of vitamin A and its precursors in the immune system. Nutr. Clin. Métab.

[19] Hernandez L.H., Hardy R.W. (2020). Vitamin A functions and requirements in fish. Aquac. Res..

[20] Liu A., To V.P.T.H., Dumas A., Hernandez J.M., Santigosa E. (2024). Vitamin nutrition in shrimp aquaculture: A review focusing on the last decade. Aquaculture.

[21] Shaik Mohamed J., Sivaram V., Christopher Roy T.S., Peter Marian M., Murugadass S., Raffiq Hussain M. (2003). Dietary vitamin A requirement of juvenile greasy grouper (*Epinephelus tauvina*). Aquaculture.

[22] Shao L., Zhu X., Yang Y., Jin J., Liu H., Han D., Xie S. (2016). Effects of dietary vitamin A on growth, hematology, digestion and lipometabolism of on-growing gibel carp (*Carassius auratus gibelio var. CAS III*). Aquaculture.

[23] Guimarães I.G., Lim C., Yildirim-Aksoy M., Li M.H., Klesius P.H. (2014). Effects of dietary levels of vitamin A on growth, hematology, immune response and resistance of Nile tilapia (*Oreochromis niloticus*) to Streptococcus iniae. Anim. Feed Sci. Technol..

[24] Huang Q., Kwaku A., Du T., Tan B., Chi S., Yang Q., Zhang S., Liu H., Dong X., Yang Y. (2018). Dietary vitamin A requirement of *Sillago sihama* Forskål. Aquac. Res..

[25] Blomhoff R., Green M.H., Green J.B., Berg T., Norum K.R. (1991). Vitamin A metabolism: New perspectives on absorption, transport, and storage. Physiol. Rev..

[26] Liñán-Cabello M.A., Paniagua-Michel J. (2004). Induction factors derived from carotenoids and vitamin A during the ovarian maturation of *Litopenaeus vannamei*. Aquac. Int..

[27] Gu P.-L., Silva Gunawardene Y.I.N., Chow B.C.K., He J.G., Chan S.-M. (2002). Characterization of a novel cellular retinoic acid/retinol binding protein from shrimp: Expression of the recombinant protein for immunohistochemical detection and binding assay. Gene.

[28] Wong W.H., Sun T., Cheung S.G., Li A.J. (2000). Effects of dietary vitamin A on phenoloxidase activity, bactericidal activity and disease resistance in Penaeus chinensis (*Crustacea: Penaeidae*). Asian Mar. Biol..

[29] Raja Gopal Reddy M., Pavan Kumar C., Mahesh M., Sravan Kumar M., Mullapudi Venkata S., Putcha U.K., Vajreswari A., Jeyakumar S.M. (2016). Vitamin A deficiency suppresses high fructose-induced triglyceride synthesis and elevates resolvin D1 levels. Biochim. Biophys. Acta Mol. Cell Biol. Lipids.

[30] Mpwaga A.Y., Gyan R.W., Ding M.Y., Tan B., Chi S., Yang Q. (2025). Effects of dietary vitamin A supplementation on growth, immune response, and lipid metabolism in mid-stage *Epinephelus coioides*. Anim. Feed Sci. Technol..

[31] Yuan H., Xie M., Hu N., Zheng Y., Hou C., Tan B., Shi L., Zhang S. (2023). Growth, immunity and transcriptome response to different stocking densities in *Litopenaeus vannamei*. Fish Shellfish Immunol..

[32] Wang T., Li W., Shan H., Ma S. (2021). Responses of energy homeostasis and lipid metabolism in *Penaeus vannamei* exposed to ammonia stress. Aquaculture.

[33] AOAC International (2023). Official Methods of Analysis of AOAC International.

[34] (2024). Determination of Vitamin A in Feeds—High Performance Liquid Chromatography.

[35] Emerenciano M., Gaxiola G., Cuzo G. (2013). Biofloc Technology (BFT): A Review for Aquaculture Application and Animal Food Industry. Biomass Now—Cultivation and Utilization.

[36] Krummenauer D., Peixoto S., Cavalli R.O., Poersch L.H., Wasielesky W. (2011). Superintensive Culture of White Shrimp, *Litopenaeus vannamei*, in a Biofloc Technology System in Southern Brazil at Different Stocking Densities. J. World Aquac. Soc..

[37] Yang Q.H., Zhou Q.C., Chi S.Y., Dong X.H. (2007). Effect of dietary vitamin A levels on growth, feed utilization, body composition and non-specific immunological responses of juvenile shrimp. Chin. J. Anim. Nutr..

[38] He H., Lawrence A.L., Liu R. (1992). Evaluation of dietary essentiality of fat-soluble vitamins, A, D, E and K for penaeid shrimp (*Penaeus vannamei*). Aquaculture.

[39] Darvishpour H., Yahyavi M., Mohammadizadeh F., Javadzadeh M. (2012). Effects of vitamins A, C, E and their combination on growth and survival of *Litopenaeus vannamei* post larvae. Adv. Stud. Biol..

[40] Gao Y., He Z., Vector H., Zhao B., Li Z., He J., Lee J.-Y., Chu Z. (2017). Effect of stocking density on growth, oxidative stress and HSP70 of Pacific white shrimp *Litopenaeus vannamei*. Turk. J. Fish. Aquat. Sci..

[41] Liu M., Chen J., Zhu W., Tan Q., Wu F., Han D. (2017). Effects of dietary vitamin A on growth performance and calcium and phosphorus contents in bone of subadult grass carp (*Ctenopharyngodon idellus*). Acta Hydrobiol. Sin..

[42] Luquet G., Marin F. (2015). Biomineralizations: Insights and prospects from crustaceans. ZooKeys.

[43] Feng D., Wang X., Li E., Bu X., Qiao F., Qin J., Chen L. (2019). Dietary Aroclor 1254-Induced Toxicity on Antioxidant Capacity, Immunity and Energy Metabolism in Chinese Mitten Crab *Eriocheir sinensis*: Amelioration by Vitamin A. Front. Physiol..

[44] Le Moullac G., Haffner P. (2000). Environmental factors affecting immune responses in crustacea. Aquaculture.

[45] Esmaeili N., Ma H., Kadri S., Tocher D.R. (2024). Protein and lipid nutrition in crabs. Rev. Aquac..

[46] Duan Y., Zhang J., Huang J., Jiang S. (2019). Effects of Dietary Clostridium butyricum on the Growth, Digestive Enzyme Activity, Antioxidant Capacity, and Resistance to Nitrite Stress of Penaeus monodon. Probiot. Antimicrob. Proteins.

[47] Schlaepfer I.R., Joshi M. (2020). CPT1A-mediated Fat Oxidation, Mechanisms, and Therapeutic Potential. Endocrinology.

[48] Li Y., De J., Jiang Q., Yang Y., Xu W., Du X., Zhao Y. (2022). Comparison of lipid metabolism between broodstock and hybrid offspring in the hepatopancreas of juvenile shrimp (*Macrobrachium nipponense*): Response to chronic ammonia stress. Anim. Genet..

[49] Moren M., Opstad I., Berntssen M., Zambonino Infante J., Hamre K. (2004). An optimum level of vitamin A supplements for Atlantic halibut (*Hippoglossus hippoglossus* L.) juveniles. Aquaculture.

[50] Napoli J.L. (2012). Physiological insights into all-trans-retinoic acid biosynthesis. Biochim. Biophys. Acta Mol. Cell Biol. Lipids.

[51] Shiau S., Chen Y. (2000). Estimation of the Dietary Vitamin A Requirement of Juvenile Grass Shrimp, Penaeus monodon. J. Nutr..

[52] Zhao W., Luo H., Zhu W., Yuan X., Shao J. (2021). Effects of Time-Dependent Protein Restriction on Growth Performance, Digestibility, and mTOR Signaling Pathways in Juvenile White Shrimp *Litopenaeus vannamei*. Front. Physiol..

[53] Saxton R.A., Sabatini D.M. (2017). mTOR Signaling in Growth, Metabolism, and Disease. Cell.

[54] Song S., Kim S., Lee Y., Kim S., Lee Y., Kim W., Kim M., Jung H., Kim D., Lee K. (2026). Dietary Leucine Requirement and Its Effects on Growth, Immunity, Antioxidant Capacity and Protein Metabolism in Pacific White Shrimp, *Penaeus vannamei*. Mar. Biotechnol..

[55] Liao X.-D., Zhou C.-H., Zhang J., Shen J.-L., Wang Y.-J., Jin Y.-C., Li S.-L. (2020). Effect of all-trans retinoic acid on casein and fatty acid synthesis in MAC-T cells. Asian-Australas. J. Anim. Sci..

[56] Kahlhofer J., Teis D. (2023). The human LAT1–4F2hc (SLC7A5–SLC3A2) transporter complex: Physiological and pathophysiological implications. Basic Clin. Pharmacol. Toxicol..

[57] Wei Z., Zhuang Y., Liu X., Zou D., Mai K., Sun Z., Ye C. (2023). Leucine promotes protein synthesis of juvenile white shrimp *Litopenaeus vannamei* through TOR signaling pathway. Aquaculture.

[58] Li E., Wang X., Chen K., Xu C., Qin J.G., Chen L. (2017). Physiological change and nutritional requirement of Pacific white shrimp *Litopenaeus vannamei* at low salinity. Rev. Aquac..

[59] Chen J., Zeng M., Liang X., Peng D., Xie R., Wu D. (2024). Dietary supplementation of VA enhances growth, feed utilization, glucose and lipid metabolism, appetite, and antioxidant capacity of Chinese perch (*Siniperca chuatsi*). Fish Physiol. Biochem..

[60] Öz Ö.B. (2024). Effects of vitamin A added to diet in different amounts on oxidative stress, antioxidant defence of soft shelled freshwater crayfish *Pontastacus leptodactylus* (Esch, 1823). J. Mater. Electron. Devices.

[61] Kloc M., Halasa M., Kubiak J.Z., Ghobrial R.M. (2024). Invertebrate Immunity, Natural Transplantation Immunity, Somatic and Germ Cell Parasitism, and Transposon Defense. Int. J. Mol. Sci..

[62] Tassanakajon A., Somboonwiwat K., Supungul P., Tang S. (2013). Discovery of immune molecules and their crucial functions in shrimp immunity. Fish Shellfish Immunol..

[63] Pope E.C., Powell A., Roberts E.C., Shields R.J., Wardle R., Rowley A.F. (2011). Enhanced Cellular Immunity in Shrimp (*Litopenaeus vannamei*) after ‘Vaccination’. PLoS ONE.

[64] Zhao M., Lin Z., Zheng Z., Yao D., Yang S., Zhao Y., Chen X., Aweya J.J., Zhang Y. (2023). The mechanisms and factors that induce trained immunity in arthropods and mollusks. Front. Immunol..

[65] Shi J., Li W., Jiang S., Diao H., Huang J., Yang L., Li Y., Ding Y., Duan Y., Chen X. (2026). Effects of Stocking Density on Growth Performance, Physiological Responses, and Transcriptomic Profile of Penaeus monodon. Int. J. Mol. Sci..

[66] Smith V.J., Brown J.H., Hauton C. (2003). Immunostimulation in crustaceans: Does it really protect against infection?. Fish Shellfish Immunol..

[67] Joza N., Susin S.A., Daugas E., Stanford W.L., Penninger J.M. (2001). Essential role of the mitochondrial apoptosis-inducing factor in programmed cell death. Nature.

[68] Sun L., Liu F., He M., Xu J., Wu C., Zhang S., Gao J., Dai J. (2025). Preserving Life: How Retinoic Acid (RA) Enhances Cell Viability and Reduces Apoptosis in Cryopreserved Blastocyst Cells of Pudong Chickens. Cells.

[69] Pettersson F., Dalgleish A.G., Bissonnette R.P., Colston K.W. (2002). Retinoids cause apoptosis in pancreatic cancer cells via activation of RAR-γ and altered expression of Bcl-2/Bax. Br. J. Cancer.

[70] Heo S., Kwak J., Jang K.L. (2015). All-trans retinoic acid induces p53-depenent apoptosis in human hepatocytes by activating p14 expression via promoter hypomethylation. Cancer Lett..

[71] Liu J., Hu Z., Guo X., Zhao L., Su C., Wang W. (2025). Identification of three genes in the methyl farnesoate signaling pathway from the Pacific whiteleg shrimp *Litopenaeus vannamei* and their roles in methyl farnesoate-mediated ovarian development. Aquac. Rep..

[72] Qian Z., He S., Liu T., Liu Y., Hou F., Liu Q., Wang X., Mi X., Wang P., Liu X. (2014). Identification of ecdysteroid signaling late-response genes from different tissues of the Pacific white shrimp, *Litopenaeus vannamei*. Comp. Biochem. Physiol. A Mol. Integr. Physiol..

[73] Hall J.A., Grainger J.R., Spencer S.P., Belkaid Y. (2011). The Role of Retinoic Acid in Tolerance and Immunity. Immunity.

[74] Arancibia S.A., Beltrán C.J., Aguirre I.M., Silva P., Peralta A.L., Malinarich F., Hermoso M.A. (2007). Toll-like Receptors are Key Participants in Innate Immune Responses. Biol. Res..

[75] Myllymaki H., Valanne S., Ramet M. (2014). The Drosophila Imd signaling pathway. J. Immunol..

